# Effete and Cullin 4 affect nuclear organization of the *gypsy* chromatin insulator

**DOI:** 10.1186/s12915-026-02596-6

**Published:** 2026-04-17

**Authors:** Shue Chen, Dagyeong Yang, Elissa P. Lei

**Affiliations:** 1 Key Laboratory of Reproductive Health Diseases Research and Translation of Ministry of Education, International Center for Aging and Cancer, Hainan Medical University, Haikou, China; 2 Program in Computational Biology, Bioinformatics, and Genomics, University of Maryland, College Park, MD USA; 3 Nuclear Organization and Gene Expression Section, Laboratory of Biochemistry and Genetics, National Institute of Diabetes and Digestive and Kidney Diseases, National Institutes of Health, Bethesda, MD USA

**Keywords:** Ubiquitination, Effete, Cullin 4, *Gypsy* insulator, 3D genome organization, TAD borders, Gene expression

## Abstract

**Backgound:**

Chromatin insulators demarcate the genome into distinct transcriptional domains and contribute to higher-order genome organization. In Drosophila, Su(Hw), CP190, and Mod(mdg4)67.2 are core protein components of the gypsy insulator complex. Multimerization of these core components contributes to the formation of large structures within the nucleus termed insulator bodies. Post-translational modifications of insulator proteins appear to affect insulator body localization and to be required for full insulator activity, but few factors involved in these processes have been identified.

**Results:**

To address this gap in understanding, we performed a high-throughput visual screen for Mod(mdg4)67.2-GFP localization using a ubiquitination-related RNAi library. We identified ubiquitination pathway proteins Effete (Eff) and Cullin 4 (Cul4), as novel regulators of CP190 localization and function. Both Eff and Cul4 physically associate with *gypsy* insulator proteins and promote *gypsy*-dependent insulator barrier activity. Moreover, Cul4 extensively colocalizes with CP190 on chromatin and assists in the recruitment of CP190 to *gypsy* sites. Both Eff and Cul4 affect transcription near topologically associating domain (TAD) borders, with Eff specifically altering the three-dimensional (3D) nuclear positioning of *gypsy* insulator sites.

**Conclusions:**

Our findings reveal a novel role for ubiquitination pathway-related enzymes in chromatin insulator activity, 3D genome organization, and gene expression.

**Supplementary Information:**

The online version contains supplementary material available at 10.1186/s12915-026-02596-6.

## Backgound

Chromatin insulators are DNA–protein complexes that control higher-order genome organization to regulate gene expression. Insulators prevent improper interactions between cis-regulatory elements and promoters, thereby promoting the organization of eukaryotic genomes into independent transcriptional domains [1–3]. Insulators also act as a barrier to prevent the spread of repressive chromatin over transcriptionally active genes. By promoting DNA looping, insulators stabilize contacts or block communication between distant sites [4]. In *Drosophila*, several zinc-finger proteins have been identified to assist the chromatin binding of the universal CP190 (Centrosomal protein 190) insulator protein [5–8]. One well-characterized insulator complex, the *gypsy* chromatin insulator (also known as the Suppressor of Hairy wing [Su(Hw)] insulator), mainly localizes to introns and intergenic regions of the genome [9, 10]. However, other DNA-binding insulator proteins such as CTCF, BEAF-32, M1BP, and ZIPIC, which also physically associate with CP190, are mostly enriched near gene promoters and TAD borders [10–12]. Previous work has shown that depletion of Su(Hw) can alter the genome-wide distribution of CP190 and lead to its accumulation at non-Su(Hw) insulator sites [13], perhaps affecting overall 3D genome organization.

The three core components of the *gypsy* insulator complex are Su(Hw), Mod(mdg4)67.2 (Modifier of mdg4 [Mod(mdg4)] 67.2 kDa isoform), and CP190. Su(Hw) recruits the other core components to chromatin and harbors a cluster of 12 zinc fingers, which dictate its DNA-binding specificity [6, 14, 15]. CP190 and Mod(mdg4)67.2 both harbor an N-terminal BTB/POZ domain and can form homo- or heterodimers [6, 16, 17]. Multimerized *gypsy* insulator proteins coalesce into discrete foci termed insulator bodies in interphase cells [18, 19]. The formation of insulator bodies can also be induced by stress, such as osmostress and cold temperature [13, 20]. While the physiological function of a visually defined structure such as the insulator body is difficult to assess, *gypsy* insulator function is highly correlated with the wild-type localization of *gypsy* insulator bodies [6, 18, 19, 21, 22]. Thus, monitoring the localization of insulator bodies can be used as a functional readout of *gypsy* insulator function.

Insulator proteins can be post-translationally modified by SUMOylation, which affects their nuclear localization and likely their function. Both CP190 and Mod(mdg4)67.2 have been shown to be SUMOylated in vitro and in vivo [23–25], but these studies arrived at opposing conclusions regarding whether this modification leads to a positive or negative effect on insulator activity. Furthermore, an E3 ubiquitin ligase, Topors (*Drosophila* Topoisomerase I-interacting RS protein) promotes the formation of insulator bodies and *gypsy* insulator enhancer-blocking activity [26]. Moreover, Topors overexpression antagonizes CP190 and Mod(mdg4)67.2 SUMOylation [25]. Topors functions through physical interaction with LaminB to tether *gypsy* insulator complexes to a nuclear substrate [26], but it is unknown whether or how ubiquitination plays a role in insulator function.

Ubiquitin transfer to the substrate is catalyzed by an E2 ubiquitin-conjugating enzyme, such as the highly conserved Eff (also known as UbcD1). *Drosophila* Eff has been found to prevent telomere fusion and repress gene expression near the telomere [27, 28]. DamID profiling in Kc167 (Kc) hemocyte cells showed that Eff preferentially associates with repressive regions in BLUE (Polycomb Group) and BLACK (low expression) chromatin, similar to Su(Hw) and LaminB [29]. Mutation of *eff* was also shown to enhance the *polycomb* phenotype of *polyhomeotic* mutants [30]. While Eff functions in ubiquitin-mediated degradation of several proteins during development and cell cycle progression through regulation of Cyclin A [27, 31–35], these factors are not chromatin related, and it remains unknown what other ubiquitination pathway-related factors may cooperate with Eff.

E2 ubiquitin-conjugating enzymes require an E3 ubiquitin ligase to transfer ubiquitin to target proteins, and E2-E3-substrate interactions can be promoted by scaffolding proteins termed Cullins. Like Eff, Cullin 4 (Cul4) is essential for proper cell cycle progression but functions through degradation of the Cdk inhibitor Dacapo and Cyclin E [36]. Moreover, highly conserved *S. pombe* and human Cul4 orthologs can alter histone methylation to regulate gene expression [37–39]. To date, no evidence shows a physical or functional interaction between Eff and Cul4.

Here, we performed a high-content RNAi imaging screen to identify ubiquitination pathway factors that affect *gypsy* insulator localization using Mod(mdg4)67.2-GFP-tagged Kc cells. We newly identified two factors, Eff and Cul4, that affect the formation of *gypsy* insulator bodies and explored whether they might cooperatively influence *gypsy* insulator function and/or 3D genome organization. We found that Eff and Cul4 physically interact with one another and CP190, and both factors promote *gypsy* insulator barrier activity. ChIP-seq profiling in Kc cells revealed extensive overlap between Cul4 and CP190 at promoters, affecting the expression of genes located near TAD borders. Finally, depletion of Eff specifically alters the 3D positioning of *gypsy* insulator binding sites, providing further evidence for the involvement of ubiquitination factors in promoting *gypsy* insulator activity, genome organization, and gene regulation.

## Results

### Eff and Cul4 affect the nuclear localization of insulator bodies and physically interact with CP190

**Fig. 1:**
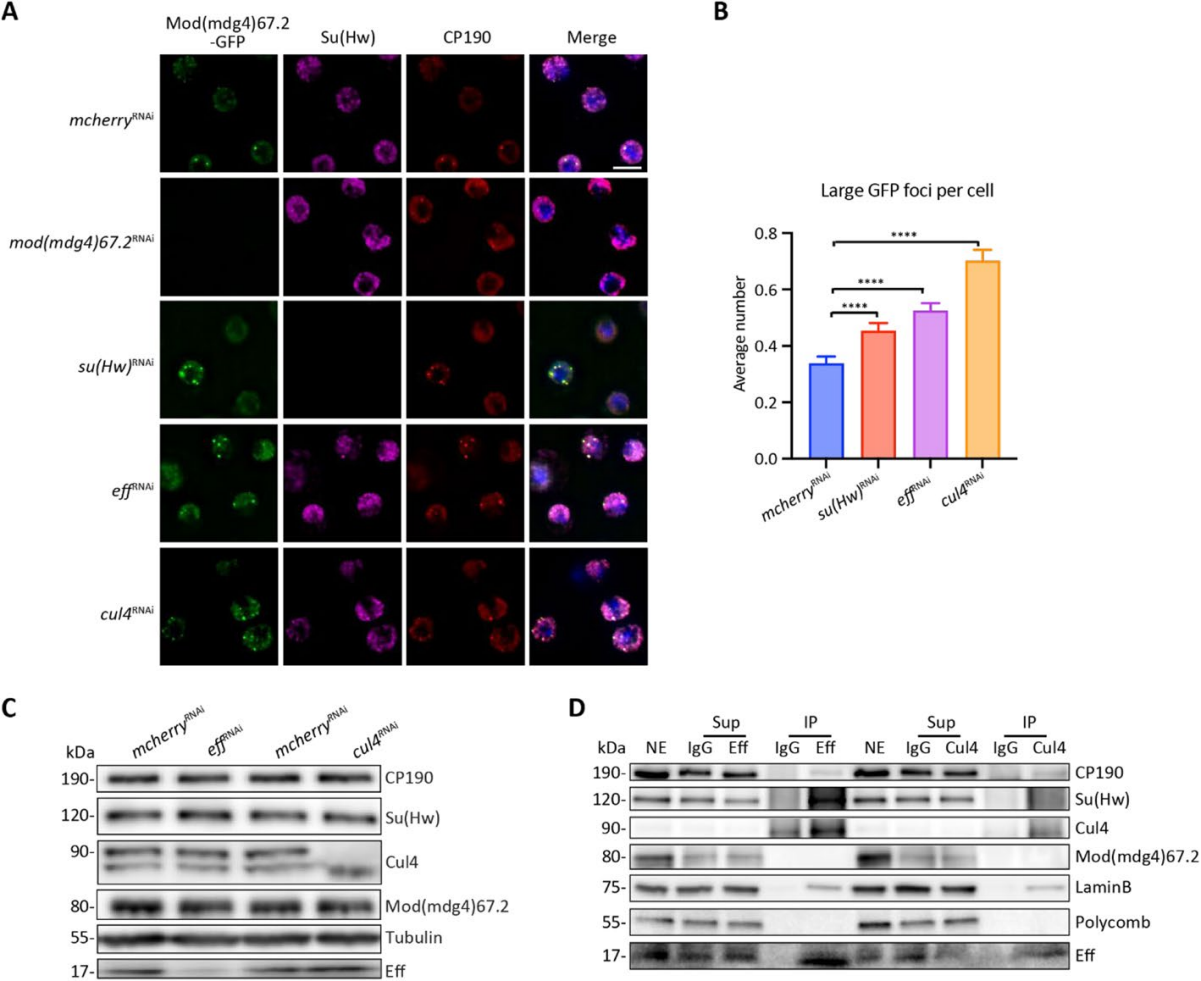
Eff and Cul4 affect the formation of *gypsy* insulator bodies and physically associate with CP190. **A** Representative images of insulator bodies marked by Mod(mdg4)67.2-GFP (green), Su(Hw) (magenta), and CP190 (red). Nuclei are marked with DAPI (blue) in the merged image. Kc cells were transfected with *mcherry* (control)*, su(Hw)*,* mod(mdg4)67.2*,* eff*, or *cul4* dsRNA and were incubated at 25 °C for 4 d before fixation and immunostaining using anti-GFP, anti-Su(Hw), and anti-CP190, respectively, on glass slides. The maximum-intensity projection of the *z*-stack is shown for visualization purposes only. Scale bars: 5 μm. **B** Quantification of the average number of large (volume > 0.5 μm^3^) GFP foci per cell. Cells knocked down with *mcherry* dsRNA were used as a negative control and compared with other dsRNA treatments. Three biological replicates and n > 500 cells for each condition were used in this assay. Data were analyzed with a two-tailed unpaired t-test, and error bars represent standard deviation. ****P < 0.0001. **C** Western blotting of total lysates from control, *eff* and *cul4* knockdown Kc cells to assess any impact on *gypsy* insulator protein levels when Eff or Cul4 is depleted in cells. Tubulin was used as a loading control. **D** Immunoprecipitation of Eff or Cul4 co-purifies CP190 and LaminB. Nuclear extracts (NE) were obtained from embryos aged 0–24 h collected at room temperature and immunoprecipitated with rabbit anti-Eff, rabbit anti-Cul4 antibody, or normal serum (IgG) as indicated. NE input, unbound supernatant (Sup) and bound (IP) fractions are shown. Polycomb is not co-purified and is shown as a negative control

Localization of *gypsy* insulator bodies has previously been used as a phenotypic readout for a high-content visual screen to identify factors that affect *gypsy* insulator activity. For example, NURF301 and Pita were identified as regulators of Mod(mdg4)67.2-GFP localization in Kc cells after knockdown using the *Drosophila* RNAi Screening Center (DRSC) transcription factor sub-library [13]. In this study, we used the same strategy to screen the ubiquitin-related DRSC sub-library, which contains 439 genes. For the screening, *mcherry* dsRNA was used as a negative control, and we also verified that *mod(mdg4)67.2* knockdown reduces the GFP signal (Additional File 1: Fig. S1). We used *su(Hw)* knockdown as a positive control, resulting in a statistically significant increase in large Mod(mdg4)67.2-GFP foci, likely due to resultant release from chromatin and increased propensity to multimerize under these conditions [13]. Two strong hits were identified in this screen, Eff, an E2 ubiquitin-conjugating enzyme, and Cul4, a scaffolding component of a Cullin-RING E3 ubiquitin ligase complex [40–42] (Additional File 1: Fig. S1 and Additional File 2: Table S1). Follow-up knockdown analysis of cells grown at room temperature and imaged on glass slides shows that knockdown of *eff* leads to an increase of large GFP foci similar to knockdown of *su(Hw)*, while *cul4* knockdown results in a similar effect (Fig. 1A–B). Importantly, no significant effect on cell viability or changes in endogenous core *gypsy* insulator protein or Mod(mdg4)67.2-GFP expression levels are observed after knockdown of either factor (Fig. 1C, Additional File 1: S2A, and Additional File 2: Table S2–3), indicating no steady-state effect on the turnover of insulator proteins. Moreover, Eff or Cul4 protein levels also show little change after the depletion of each other (Fig. 1C and Additional File 1: S2A-B). Although Eff and Cul4 are both implicated in Cyclin E degradation [35, 36], we did not observe any G_1_ cell cycle arrest after knockdown of *eff* or *cul4* (Additional File 1: Fig. S2C) in contrast to previous studies using higher levels of dsRNA [36, 43]. Previous studies have shown that SUMO and Topors both affect the formation of insulator bodies in interphase cells [23, 25, 26]. In our screen, s*mt3*, which encodes SUMO, was also identified as a hit, but its depletion resulted in high levels of cell death and was therefore not further pursued (Additional File 2: Table S2). Furthermore, no change in large GFP foci or *gypsy* insulator protein levels was observed after depletion of Topors in Kc cells (Additional File 1: Fig. S2D–F). Based on these results, we determined that Eff and Cul4 affect the localization of *gypsy* insulator bodies, and Topors does not function similarly to Eff or Cul4 with respect to Mod(mdg4)67.2-GFP localization.

We next assessed whether Eff or Cul4 may physically interact with *gypsy* insulator core components in vivo. We examined previous mass spectrometry analysis of immunoaffinity purifications of either anti-CP190 or anti-Su(Hw) and found that Eff and Cul4 are indeed co-purified at low levels (Additional File 2: Table S4–5) [44]. To validate these results, we performed anti-CP190 purification from *Drosophila* embryonic nuclear extracts and confirmed the presence of Eff and Cul4 by Western blotting, and these factors are not substantially associated with normal IgG (Additional File 1: Fig. S2G). We also found that CP190, Su(Hw), and Cul4 are immunopurified using anti-Eff at a higher level than with normal IgG (Fig. 1D). Moreover, Eff and CP190 are also present specifically in anti-Cul4 immunoprecipitates. Interestingly, Eff and Cul4 also co-purify LaminB but not Mod(mdg4)67.2. As a negative control, Polycomb was not co-immunoprecipitated with any of these antibodies. These results support physical interaction between Eff and Cul4 and their association with CP190 and Su(Hw) at sub-stoichiometric levels in nuclear extracts.

### Eff and Cul4 promote *gypsy* insulator barrier function

**Fig. 2:**
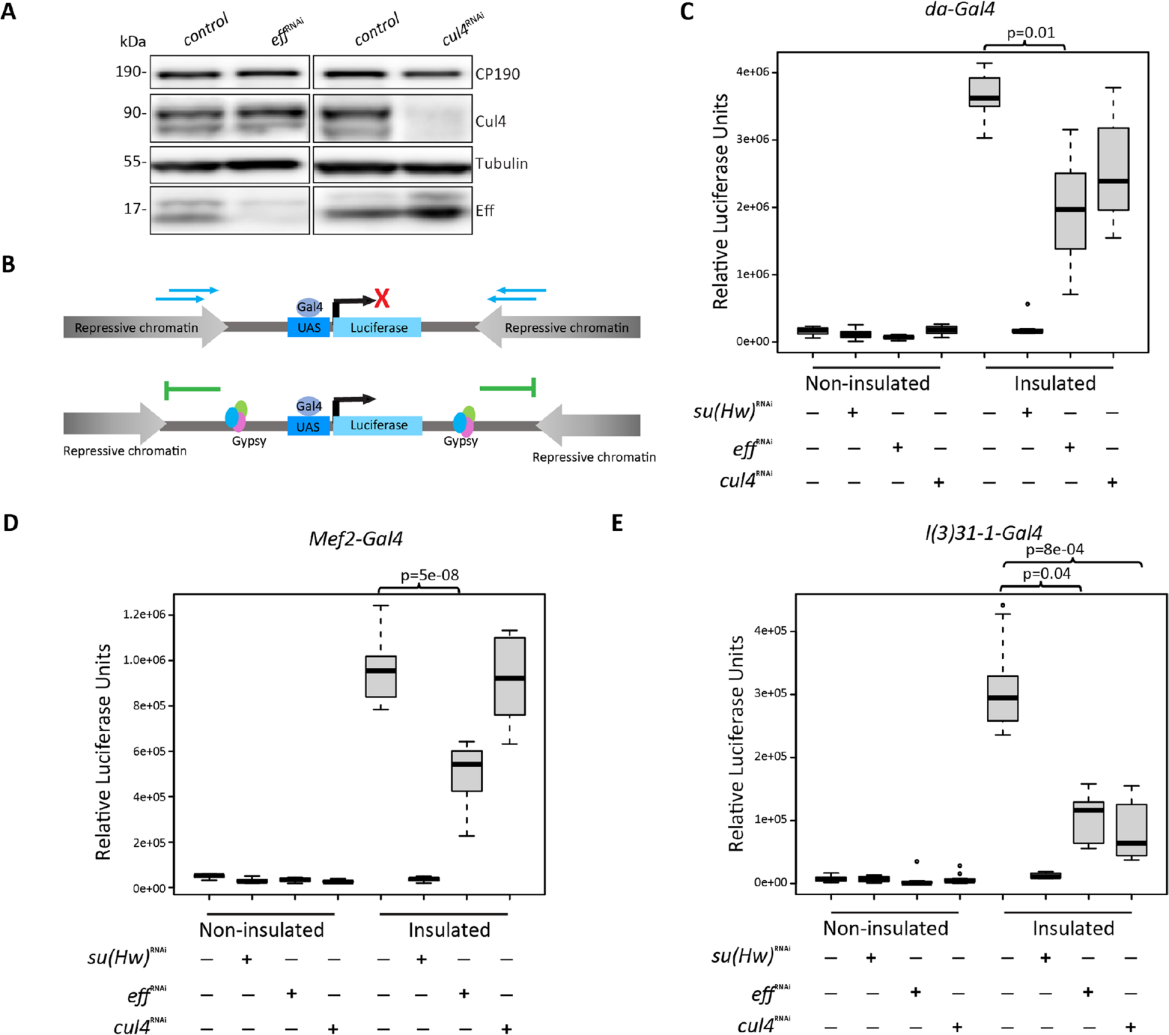
Eff and Cul4 promote *gypsy* insulator barrier activity. **A** Western blotting of second instar larval extracts for Eff, Cul4, CP190, and Tubulin as a loading control in control, *eff*, and *cul4* RNAi knockdown flies using the ubiquitously expressed *Act5C*-Gal4 driver. **B** Schematic diagram of in vivo* gypsy*-dependent insulator barrier assay. The spreading of repressive chromatin in the non-insulated UAS-luciferase line reduces luciferase expression, while the presence of the *gypsy* insulator in the insulated line acts as a barrier to allow luciferase expression. **C**–**E** Relative luciferase activity of insulated or non-insulated male third instar larvae of control, *eff*, or *cul4* RNAi driven by **C** *da*-Gal4 driver, **D** *Mef2*-Gal4 driver, and **E** *l(3)31–1*-Gal4 driver. *n* = 12 individual larvae in each genotype, and two independent experiments were performed with similar results. Data were analyzed with one-way ANOVA followed by Tukey HSD post hoc test to obtain *p*-values for pairwise comparisons. The box represents the 25–75th percentiles with the median indicated, and the whiskers show the minimum and maximum values. All comparisons are shown in Additional File 2: Table S6

Next, we explored whether Eff and Cul4 play a role in *gypsy* insulator function. To verify knockdown efficiency in vivo, we used the ubiquitously expressed *Act5C-Gal4* driver to express an RNAi hairpin targeting *eff* or *cul4*, respectively, and performed western blotting of second instar larvae (Fig. 2A). Consistent with previous studies, ubiquitous depletion of Eff or Cul4 using *Act5C-Gal4* leads to 100% lethality by the third instar larval stage [27, 45]. Therefore, we performed ubiquitous but lower-expression knockdown using *da-GAL4*, allowing development to the pupal stage but not adult eclosion.

We examined the effect of Eff and Cul4 on *gypsy*-dependent barrier activity in various tissues of third instar larvae. In this assay, an upstream activation site (UAS)-driven luciferase reporter, inserted into the transcriptionally repressed *attP3* genomic site, is either insulated by flanking Su(Hw) binding sites or not insulated (Fig. 2B). A tissue-specific Gal4 driver controls both the expression pattern of the luciferase reporter and specific knockdown of the *trans*-acting candidate of interest [22]. Ubiquitous depletion of Su(Hw) using *da-*Gal4 results in loss of barrier function and a significant decrease in luciferase expression specifically in the insulated line. Moreover, depletion of Eff driven by *da-*Gal4 shows a significant reduction in luciferase expression compared to the control insulated line (Fig. 2C, Additional File 2: Table S6). Depletion of Eff using muscle-specific *Mef2-*Gal4 driver or CNS-enriched *l(3)31–1-*Gal4 driver also leads to statistically significant decreases in luciferase activity compared to controls, indicating that Eff promotes *gypsy* insulator-dependent barrier activity in a variety of tissues (Fig. 2D–E, Additional File 2: Table S6). Interestingly, reduction of luciferase activity was observed only when Cul4 is depleted using the CNS and salivary gland-specific *l(3)31–1-*Gal4 driver (Fig. 2E). We performed quantitative ChIP-PCR for CP190 and Su(Hw) at the transgene *gypsy* elements and endogenous binding sites and validated strong binding surrounding the reporter (Additional File 1: Fig. S3 and Additional File 2: Table S7). In *cul4*^*RNAi*^ larvae driven by ubiquitous *da*-Gal4, CP190 and Su(Hw) binding were mildly increased whereas both factors were mildly decreased in *eff*^*RNAi*^ larvae. Contrasting results perhaps reflect the tissue-specific nature of functional effects of *cul4*^*RNAi*^. Interestingly, we found that association of Histone H3 was greatly reduced by both knockdowns at all sites tested, suggesting that chromatin compaction may be disrupted by depletion of Cul4 and Eff. This quantitative barrier assay shows that Eff is required for *gypsy* insulator function in all tissues tested, whereas Cul4 regulates barrier activity in a tissue-specific manner.

### Cul4 colocalizes with CP190 genome-wide

**Fig. 3:**
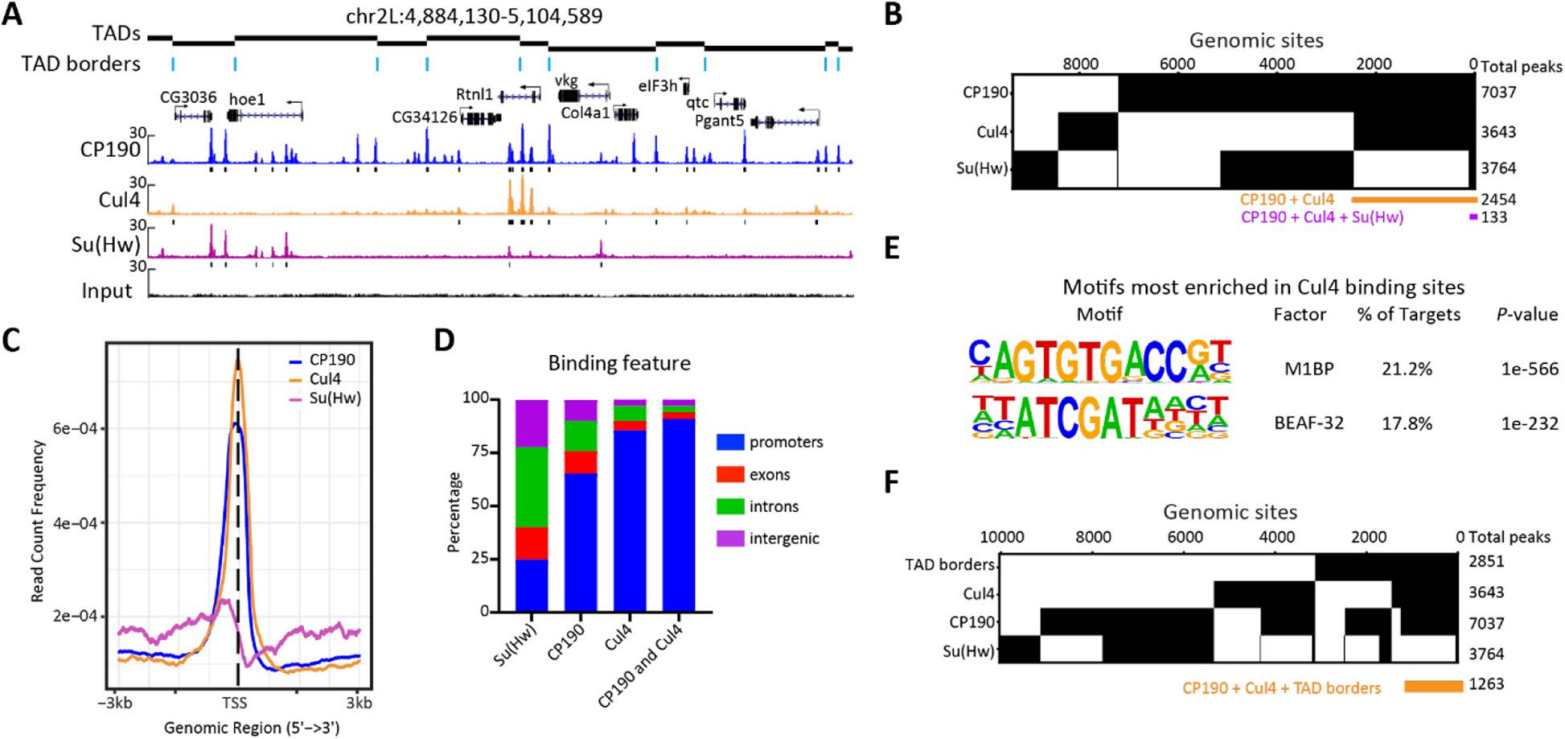
Cul4 colocalizes extensively with CP190 and binds chromatin at TAD borders. **A** Screenshot example of ChIP-seq profiles of CP190, Cul4, Su(Hw), and the input from Kc cells. Three biological replicates were performed for each sample, and peaks called by MACS2 are indicated by black bars. TADs and borders are indicated (top). **B** Binary heatmap of CP190, Cul4, and Su(Hw) binding sites in Kc cells ordered by supervised hierarchical clustering. For each factor, overlapping peaks among replicates were used. The total number of peaks of each factor is shown on the right. Each column represents a single genomic location, with the yellow bar indicating the overlapping sites of CP190 and Cul4, and the purple bar shows the colocalizing sites of CP190, Cul4, and Su(Hw). 2454 (67% of 3643) Cul4 peaks are shared with CP190, and 146 (4%) Cul4 sites overlap with Su(Hw). **C** Average signals of CP190 and Cul4 peaks, but not of Su(Hw), accumulate at the TSS. Data were collected from signals ± 3 kb of the TSS. **D** Bar plot showing the distribution of CP190, Cul4, Su(Hw), and overlapping sites of CP190/Cul4 with respect to genomic features. CP190, Cul4, and their overlapping sites are enriched at promoters, while Su(Hw) sites are mainly distal to promoters. **E** Motif enrichment of Cul4 binding sites in the genome. **F** Cul4 and CP190 are enriched at TAD borders. Binary heatmap of TAD borders, Cul4, CP190, and Su(Hw)*.* The total number of peaks is shown on the right, with overlapping sites indicated by the yellow bar

To assess the potential interaction of Eff and Cul4 with *gypsy* insulator proteins on chromatin, we performed ChIP-seq analysis for Eff, Cul4, CP190, and Su(Hw) in Kc cells. We were unable to successfully perform ChIP with anti-human Eff antibodies, despite their prior validation for Western blotting (Figs. 1C and 2A). However, we attempted Cul4 ChIP using two independent sources of anti-human Cul4 antibodies and identified 3643 Cul4 peaks (Additional File 1: Fig. S4). We found that 2624 (72%) Cul4 peaks were reduced after depletion of Cul4 using DiffBind3 analysis, verifying the specificity of the antibody used for further analysis. We also identified 7037 CP190 and 3764 Su(Hw) peaks in the genome, with 2454 genomic sites (67% of Cul4 peaks) shared between CP190 and Cul4 (Fig. 3A–B). Only 146 Su(Hw) sites overlap with Cul4 (4% of Cul4 peaks), and among those, 133 sites are also occupied by CP190 (Fig. 3B). Moreover, CP190 and Cul4 are mostly enriched at transcription start sites (TSS), while Su(Hw) is primarily located in introns and intergenic regions (Fig. 3C–D). Overall, these results indicate extensive colocalization of Cul4 with CP190, particularly in promoter regions. We performed motif analysis of Cul4 binding sites using the HOMER algorithm and found enrichment of M1BP and BEAF-32 binding motifs, factors that are enriched at TAD boundaries (Fig. 3E). Note that the binding motifs of BEAF-32 and DREF are nearly identical [46]. We also examined Cul4 enrichment near TAD boundaries. Approximately 51% of TAD boundaries harbor Cul4 binding, the majority of which are also occupied by CP190 (44% of total TAD boundaries) (Fig. 3F, Fisher’s exact test (FET), *P* < 2.2e − 16, odds ratio = 4.0). Our results indicate that, like CP190, Cul4 binds near TSSs and may affect gene regulation.

### Eff and Cul4 regulate transcription of an overlapping gene set near TAD borders

**Fig. 4:**
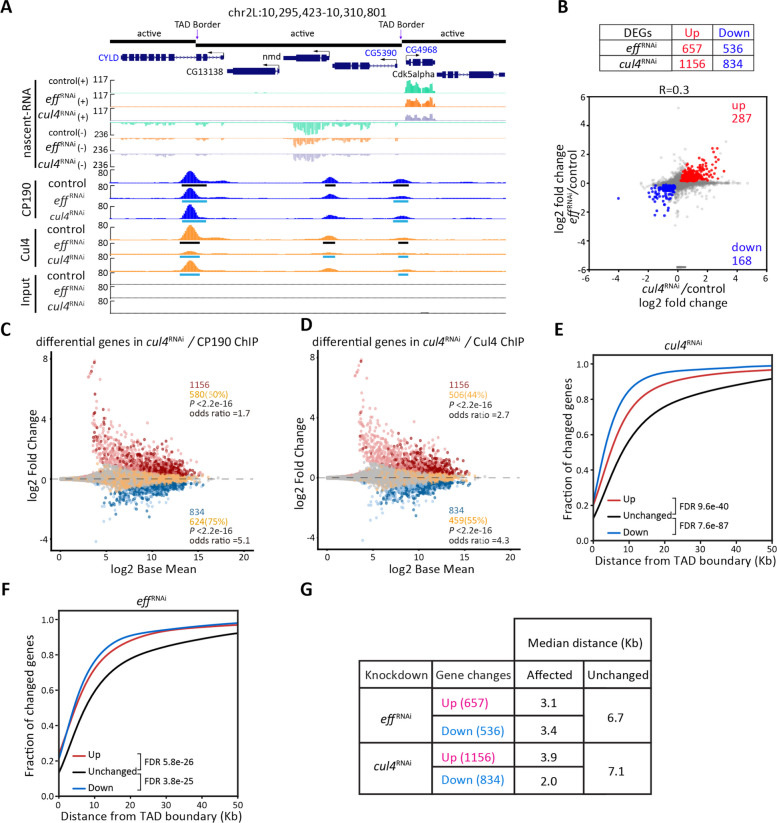
Eff and Cul4 transcriptionally regulate similar gene sets near TAD borders. **A** Example screenshot showing promoter association of CP190 and Cul4 at differentially expressed genes (DEGs) of neuRNA-seq after depletion of Eff or Cul4 in Kc cells. CP190 and Cul4 are enriched at promoters of genes located near TAD borders. Down-regulated genes are labeled in blue, and unaffected are labeled in black. Vertical arrows indicate TAD borders. Peaks called by MACS2 in control samples are indicated by black rectangles, with decreased peaks indicated with blue in knockdown conditions. The DiffBind algorithm was used to determine differential peaks, and three biological replicates were used for each condition (FDR < 0.05). **B** The table (top) shows the number of DEGs in neuRNA-seq analyzed by DEseq2. The scatter plot (bottom) compares neuRNA-seq profiles of *eff* and *cul4* RNAi to the control. Pearson’s R corresponds to the correlation coefficient between the two profiles. Common up-regulated genes are indicated in red, and common down-regulated genes are indicated in blue. **C**–**D** MA plots indicating neuRNA-seq affected genes after depletion of Cul4. Statistically significant changes include 1156 up-regulated genes (red) and 834 down-regulated genes (blue) using *P*_adj_ < 0.05. Unchanged genes without CP190 (**C**) and Cul4 (**D**) binding are indicated in gray. Promoters of unchanged genes containing CP190 or Cul4 peaks are colored in orange. Up-regulated genes with (dark red) or without (light red) peak at the promoter, and down-regulated genes with (dark blue) or without (light blue) binding at the promoter are shown. Two-sided Fisher’s exact test was used to obtain the *P*-value and odds ratio of CP190 or Cul4 binding at the promoter of affected genes compared to unchanged genes. **E**–**F** Cumulative histograms showing promoter distance from the closest TAD border classified by change in nascent expression in *cul4* (**E**) or *eff* (F) knockdown cells. Upregulated (red), downregulated (blue) or unchanged (black) genes are indicated. **G** The summary table shows the median distance from the closest TAD border to up- and down-regulated genes, respectively, compared to unchanged in either knockdown condition. FDR was determined by Mann–Whitney U test

As Cul4 is greatly enriched at TSSs, we performed nascent EU (neuRNA)-seq to examine newly synthesized transcripts in the *cul4* RNAi knockdown condition to determine whether Cul4 affects transcription genome-wide. Depletion of Cul4 results in up-regulation of 1156 genes and down-regulation of 834 genes (Fig. 4A–B). In Eff-depleted cells, 657 up-regulated genes and 536 down-regulated genes were identified. We found that 38% of differentially expressed genes (DEGs) in *eff* knockdown cells were also significantly affected after depletion of Cul4 (Fig. 4B). Because of the smaller fold change and number of differential genes in *eff* knockdown cells, the Pearson correlation score is positive but not high (*R* = 0.3) (Fig. 4B). These results indicate that the impact of Eff on transcription is milder compared to Cul4, even though they regulate gene sets that are partially overlapping.

Given that chromatin binding of both Cul4 and CP190 is enriched at promoters, we quantified the number of DEGs in *cul4*-depleted cells that harbor binding of CP190 or Cul4 at the promoter. Using ChIP-seq peaks in the control condition, we found that 50% of promoters of up-regulated genes (Fig. 4C, FET, *P* < 2.2e − 16, odds ratio = 1.7) and 75% of promoters of down-regulated genes harbor CP190 chromatin binding (Fig. 4C, FET, *P* < 2.2e − 16, odds ratio = 5.1). Similarly, Cul4 binding is highly enriched at promoters of DEGs that are up-regulated (Fig. 4D, 44%, FET, *P* < 2.2e − 16, odds ratio = 2.7) or down-regulated (Fig. 4D, 55%, FET, *P* < 2.2e − 16, odds ratio = 4.3). We performed the same analysis in *eff* knockdown cells and found that CP190 and Cul4 are also highly enriched at DEG promoters after Eff depletion (Additional File 1: Fig. S5A–B). In contrast, Su(Hw) binding is not enriched at either up-regulated or down-regulated genes in either *cul4* or *eff* knockdown cells (Additional File 1: Fig. S5C–D). Taken together, these results suggest the possibility that Cul4 and CP190 directly regulate transcription at the promoter, and Eff-dependent regulation of gene expression might involve Cul4 and CP190.

Given that Cul4 and CP190 associate with chromatin near TAD borders, we examined whether Cul4 or Eff regulates the expression of genes in the proximity of TAD borders genome-wide. The median distance between *cul4* knockdown DEG promoters and the nearest TAD border is 2.0 kb and 3.9 kb for downregulated and upregulated genes, respectively (Fig. 4E and G). These distances are significantly less than that of the unchanged genes (7.1 kb) after depletion of Cul4. Furthermore, *eff* knockdown DEG promoters are also significantly closer to TAD borders (3.4 kb and 3.1 kb for down and upregulated, respectively) than those of unchanged genes (6.7 kb) (Fig. 4F–G). Overall, our observations indicate that Eff and Cul4 affect the expression of an overlapping gene set near TAD borders genome-wide.

### Depletion of Eff or Cul4 affects chromatin binding of CP190 at differentially expressed genes

**Fig. 5:**
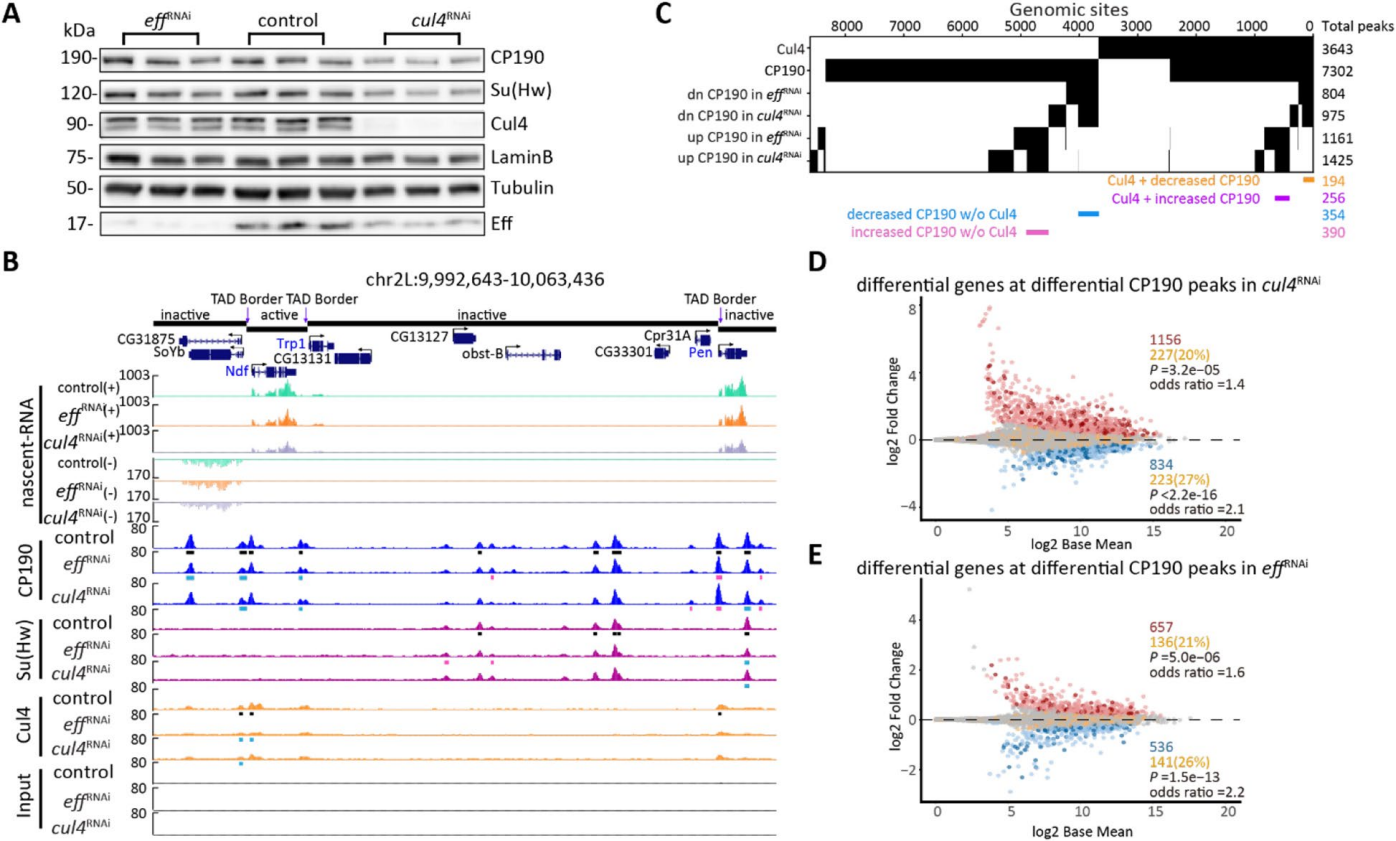
Eff and Cul4 affect chromatin binding of CP190 at differentially expressed genes. **A** Western blotting of total Kc cell lysates to detect the knockdown efficiency of Eff and Cul4 and validate no change of *gypsy* insulator protein levels. LaminB levels also show no change after depletion of Eff or Cul4. Tubulin is a loading control. **B** Screenshot example of ChIP and neuRNA-seq tracks in control, *eff*, and *cul4* RNAi cells. Peaks called by MACS2 in control samples are indicated by black rectangles, with decreased and increased peaks indicated with blue and pink, respectively, in knockdown conditions. **C** Binary heatmap of CP190, Cul4, and differentially bound peaks of CP190 in knockdown conditions. Rectangles below indicate the overlapping peaks of both decreased and increased CP190 after the depletion of Eff and Cul4 with or without colocalization of Cul4. CP190 depends on Cul4 to be recruited into these sites (FET, *P* = 0.02, odds ratio = 1.2, see materials and methods for details of FET analysis). **D**–**E** MA plots show enrichment of differential binding of CP190 at promoters of dysregulated genes of neuRNA-seq in *cul4* (**D**) and *eff* (**E**) knockdown cells. Unchanged genes with (orange) or without (gray) CP190 binding at the promoter are shown. Up-regulated genes with (dark red) or without (light red) CP190 binding, and down-regulated genes with (dark blue) and without (light blue) CP190 binding at the promoter are also shown

To determine whether Eff and Cul4 affect the recruitment of *gypsy* insulator proteins to chromatin, we performed ChIP-seq to detect core *gypsy* components after Eff or Cul4 depletion. Interestingly, we observed that Eff depletion reduces the chromatin binding of Cul4 without substantially affecting overall Cul4 protein levels (Fig. 5A-B, Additional File 1: Fig. S6A-B). Average ChIP-seq signals of Su(Hw) and CP190 are not greatly affected across the genome after knockdown of *eff* or *cul4* (Additional File 1: Fig. S6C–D). However, using the DiffBind3 algorithm, we found that after Eff depletion, 804 (11%) CP190 sites were significantly decreased, while 1161 (16%) CP190 peaks were increased (false discovery rate (FDR) < 0.05). Similarly, Cul4 depletion resulted in 975 (14%) CP190 decreased sites and 1425 (20%) CP190 increased sites. We observed that the affected CP190 peaks in either *eff* or *cul4* knockdown cells overlap considerably (Fig. 5C). Similar effects observed for CP190 binding in either knockdown are consistent with loss of Cul4 chromatin association after Eff depletion. Motif enrichment analysis indicates that decreased CP190 peaks after Cul4 or Eff depletion are enriched for the BEAF-32 and M1BP motifs, which are present at promoter regions near TAD borders [12] (Additional File 1: Fig. S6E–F). After depletion of Cul4, increased CP190 sites exhibit enrichment of the Su(Hw) binding motif (Additional File 1: Fig. S6E). Moreover, there is a substantial overlap of increased CP190 binding with Su(Hw) binding sites present in the control condition (Additional File 1: Fig. S6G). This result is consistent with a scenario in which some CP190 is released from chromatin, and a subset of this pool of CP190 is redistributed to Su(Hw) sites. In support of the possibility that changes in CP190 binding after Cul4 depletion result in changes in transcription, we found that 227 (20% of 1156) up-regulated and 223 (27% of 834) down-regulated genes after Cul4 depletion have a differential CP190 peak at their promoter (Fig. 5D). In *eff*-depleted cells, 136 (21% of 657) up-regulated and 141 (26% of 536) down-regulated genes also have a differentially bound CP190 peak at their promoters (Fig. 5E). In contrast, no enrichment of differentially bound Su(Hw) is observed for Cul4- or Eff-dependent genes (Additional File 1: Fig. S6H–I). Our results suggest that Eff and Cul4 might regulate gene expression by affecting chromatin distribution of CP190 in the genome.

### Eff specifically affects the 3D organization of *gypsy* insulator binding sites

**Fig. 6:**
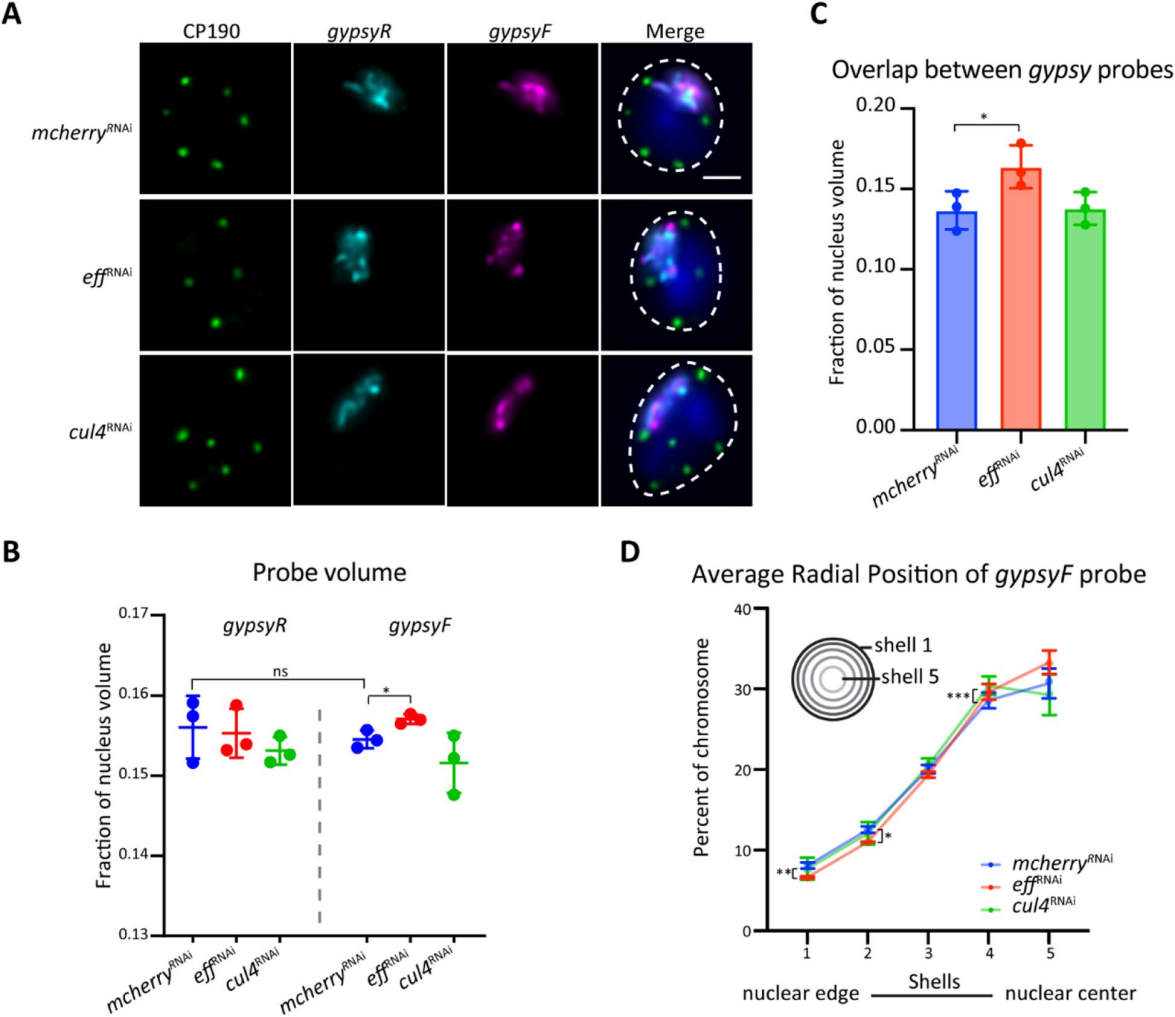
Depletion of Eff specifically alters the 3D structure of *gypsy* insulator binding sites. **A** Representative images of CP190 IF (green) and signals of *gypsyR* (cyan) and *gypsyF* (magenta) paints in RNAi-treated Kc cells. Images are maximal projections of 46 *Z*-stacks for visualization purposes only. The nuclear edge is indicated with a dashed line, and the blue signal represents DAPI staining. Scale bar: 2 µm. **B** Probe volume relative to the fraction of nuclear volume. The left panel shows the control *gypsyR* paint, the right is the *gypsyF* probe. **C** Overlapping volume of *gypsyR* and *gypsyF* paints relative to nuclear volume. Each dot represents one single replicate. *n* > 300 cells for each sample, and data are from three biological replicates. Paired *t*-test was used, and error bars show standard deviation. “ns” denotes not significant; *P < 0.05 as indicated. **D** Radial position of *gypsyF* paint in the nucleus determined by shell analysis. The nucleus was divided into five shells of equal volume, where shell 1 is the closest to the periphery, and shell 5 is the center. *n* > 500 cells. * *P* < 0.05, **P < 0.01, ***P < 0.001. Data are from three biological replicates, plotted by medians and analyzed by paired *t*-test, and error bars represent standard deviation

Above, we showed that depletion of Eff or Cul4 alters insulator body localization and mildly affects chromatin binding of CP190. We next assessed whether these two factors affect the overall 3D-spatial organization of *gypsy* DNA binding sites using Oligopaint DNA FISH. We employed “*gypsyF*” probes to detect all DNA sequences co-occupied by Su(Hw), CP190, and Mod(mdg4)67.2 on Chr3L, while a control set (reversed 1D sequences non-overlapping *gypsy* sites) of “*gypsyR*” probes detects non-*gypsy* insulator sequences with a similar size distribution [13] (Fig. 6A). We first quantified the volume of *gypsyF* and *R* paints relative to nuclear volume in RNAi-treated cells. Nuclear volume remains unaffected by any dsRNA treatments compared to *mcherry* control (Additional File 1: Fig. S7A). In control cells, the volumes of *gypsyF* and *gypsyR* paints were similar (Fig. 6B, Additional File 1: Fig. S7B–E), and no significant change is observed in *gypsyR* paint volume in either Eff or Cul4-depleted cells. However, a significant increase in *gypsyF* paint volume is specifically observed after depletion of *eff* but not *cul4* knockdown (Fig. 6B). We also found that overlap between *gypsyF* and *R* paints significantly increases after depletion of Eff (Fig. 6C). Lastly, we analyzed the nuclear position of both *gypsyF* and *gypsyR* paints in *eff*, *cul4*, and *mcherry* knockdown cells. To quantify the shift in radial positions of *gypsyF* and *gypsyR* paints in the nucleus, we performed a quantitive 3D shell analysis to examine the percentage of total signal in each concentric shell (Fig. 6D, Additional File 1: Fig. S7F). In control cells, the *gypsyF* and *gypsyR* paints extended across all shells, with most signal in the central shell (shell 5). However, in Eff-depleted cells, both *gypsyF* and *gypsyR* paints repositioned in that there was a decrease in signal near the nuclear periphery (shells 1 and 2) coupled with an increase in signal at the nuclear center (shells 4 and 5) compared to the *mcherry* control. Conversely, no significant change in signal distribution is observed after depletion of Cul4 (Fig. 6D, Additional File 1: Fig. S7F). These results suggest that depletion of Eff specifically alters the 3D position of *gypsy* insulator binding sites relative to non-*gypsy* sites, and the nuclear position of the entire chromosome shifts towards the nuclear interior.

## Discussion

In this study, we identified and explored the roles of two ubiquitin factors, Eff and Cul4, in regulating *gypsy* insulator activity and transcriptional control at TAD borders. Our results demonstrate that Eff and Cul4 are required for the proper formation of *gypsy* insulator bodies and may exert regulatory effects on gene expression near TAD borders. Additionally, we found that Eff and Cul4 associate physically with CP190, promoting *gypsy* insulator function. Eff affects chromatin binding of Cul4, and genome-wide analysis revealed extensive colocalization of Cul4 with CP190 at promoters, particularly at TAD borders. Furthermore, Eff and Cul4 mildly affect CP190 association with chromatin, resulting in its redistribution. Finally, Eff specifically alters the 3D arrangement of *gypsy* sites as well as the positioning of chromatin within nuclei. Taken together, these findings reveal the importance of Eff and Cul4 in *gyspy*-dependent insulator activity, 3D genome organization, and transcription regulation.

Previous studies showed that Eff and Cul4 are both ubiquitination-related factors involved in similar developmental pathways, but these two factors have never been studied in relation to one another. Our co-IP data show that Eff and Cul4 associate physically, although this interaction may be indirect. Both Eff and Cul4 also co-IP in substoichiometric amounts with CP190 and Su(Hw) but not Mod(mdg4)67.2, raising the possibility of physical interaction with a non-canonical insulator complex. Using DamID profiling and immunostaining, Eff was previously shown to associate with repressive regions of chromatin, similar to Su(Hw) and LaminB [29]. We were unable to verify these patterns of Eff chromatin association by ChIP-seq, but we did observe LaminB co-IP with either Eff or Cul4 antibodies. By generating the first binding profile of Cul4 in the genome, we observed high overlap with CP190 chromatin association but minimal overlap with Su(Hw). As a backbone of Cullin-RING E3 ubiquitin ligase (CRL) complexes, Cullins rely on F-box proteins or proteins containing a BTB domain or WD40 repeats to dictate substrate specificity [47]. Thus, it is possible that Cul4 binds the BTB domain of CP190 and that this interaction is incompatible with CP190 BTB domain heterodimerization with Mod(mdg4)67.2 [6].

Analysis of ChIP-seq profiles of CP190, Su(Hw), and Cul4 after depletion of Eff or Cul4 further strengthens the functional connection between Eff and Cul4 as well as their relationship with insulator proteins. Depletion of Eff results in extensive loss of Cul4 chromatin association but minimal change in overall Cul4 levels. Depletion of Cul4 decreases CP190 chromatin association at a subset of sites that appear to redistribute to Su(Hw) binding sites. Su(Hw) binding itself is also increased at these sites, suggesting that increased CP190 may help stabilize Su(Hw) binding at these locations. Moreover, CP190 is freed from promoter regions of differentially expressed genes after depletion of Eff or Cul4, and these affected genes are located near TAD borders. Given that Motif1 is enriched at CP190 sites that are lost after Eff or Cul4 depletion, one possibility is that in some manner, ubiquitination promotes CP190 association with the transcription factor M1BP at TAD borders.

Interestingly, after depletion of Eff, the 3D volume of *gypsy* binding sites changes as well as their position relative to non-*gypsy* sites. Furthermore, in Eff depleted cells, we also observed relocation of the entire chromosome towards the interior of the nucleus. However, Cul4 depletion has no effect on 3D positioning of *gypsy* or non-*gypsy* sites. Given that Cul4 does not appear to colocalize with Su(Hw), LaminB, or repressed regions in chromatin as has been reported for Eff, control of *gypsy* binding site 3D localization may reflect a Cul4-independent function of Eff. Finally, Su(Hw) and CP190 are not thought to be direct substrates of ubiquitination [25]; therefore, further studies are necessary to reveal the precise mechanistic relationship among Eff, Cul4, and insulator proteins.

## Conclusions

Our study reveals the ubiquitination-related factors Eff and Cul4 as regulators of *gypsy* insulator function and gene expression near TAD borders. We demonstrate that Eff and Cul4 physically interact, and that both associate particularly with CP190 to promote proper insulator body formation and barrier activity. Genome-wide profiling reveals extensive colocalization of Cul4 with CP190, especially at promoters and TAD borders. Depletion experiments indicate that Eff is required for Cul4 chromatin association, while Cul4 contributes to stabilizing CP190 occupancy at a subset of genomic sites. Notably, Eff modulates the 3D positioning of *gypsy* insulator sites and overall chromosome localization within the nucleus, suggesting that Eff also exerts Cul4-independent functions in higher-order genome architecture.

## Methods

### *Drosophila* strains

Flies were maintained on standard cornmeal medium at 25 °C. We used fly lines expressing dsRNA against *su(Hw)* (10724 GD), *eff* (26011 GD) and *cul4* (44829 GD) from the Vienna Drosophila RNAi Center. *Act5C-Gal4*, *da-Gal4*, *Mef2-Gal4,* and *l(3)31–1-Gal4* driver lines were obtained from the Bloomington Drosophila Stock Center. *Gypsy*-insulated *UAS-luciferase* constructs were inserted into the *attP3* landing site using *phiC31* site-specific integration, and the insertion of *attP3* in the dm6 reference genome is between 20,330,574 and 20,330,575 on chrX [48]. 100% lethality was observed in third-instar larvae when Eff or Cul4 RNAi was driven by *Act5C-Gal4* and in pupae when Eff was depleted using *Ser-Gal4*. Protein extracts from second-instar larvae were used for western blotting.

### Cell lines

Wild-type and GFP-tagged Kc cells were grown in CCM3 media (Thermo Scientific HyClone) and maintained in a monolayer at 25 °C. Mod(mdg4)67.2-GFP Kc167 cells were previously generated [13]. Briefly, cells were generated by stable random integration via plasmid transfection. A single clone was used in screening and subsequent analyses.

#### dsRNA knockdowns

DsRNA primers against *eff*, *cul4*, and *Topors* were designed based on recommendations from the *Drosophila* RNAi Screening Center (DRSC). PCR amplification was performed using primers containing the T7 promoter sequence (listed in Additional File 2: Table S8) and a cDNA template from Kc cells. Subsequently, dsRNAs were synthesized using the MEGAscript T7 in vitro transcription kit (Ambion) and purified on NucAway spin columns (ThermoFisher). Seven × 10^6^ Kc cells were transfected with 5 µg of dsRNA against *eff*,* cul4*,* Topors*,* Cp190*,* su(Hw)*,* mod(mdg4)*67.2 isoform, or *mcherry* using the Amaxa Nucleofector kit V and electroporated using the G-30 program in a 4D-Nucleofector (Lonza). Cells were incubated for 4 d at 25 °C before harvest, and knockdown efficiency was validated by western blotting and immunostaining.

### Immunofluorescence

Cells were centrifuged at 500 × *g* for 5 min and washed once in PBS. 10^5^ cells were dispensed onto Poly-L-Lysine-coated slides (Electron Microscopy Sciences) at RT for 10 min and fixed in 4% paraformaldehyde (PFA, EMS) in PBS at RT for 10 min. After two washes with PBS, cells were permeabilized with 0.5% Triton X-100 for 10 min and washed again. Next, cells were blocked in 5% bovine serum albumin (BSA, Sigma) in PBST (0.1% Triton X-100 in PBS) for 30 min before incubation with primary antibody for 1 h. Cells were washed 3 times with PBST and then incubated with secondary antibodies for 1 h and washed again. Finally, cells were stained for 10 min in 2 μg/mL 4′,6-diamidino-2-phenylindole (DAPI, Molecular Probes) prepared in PBS and mounted using ProLong Diamond (Life Technologies). For IF/FISH, slides were fixed with 4% PFA for 10 min before proceeding with FISH as described below.

### RNAi screening

The sub-library of ubiquitin-related genes (DRSC 2.0) was purchased from DRSC at Harvard Medical School. It is comprised of 6 × 384-well plates, containing 439 genes and 2–3 pairs of dsRNAs for each gene in independent wells. We performed the screen as previously described [13]. We used *mcherry* knockdown as a negative control, *su(Hw)* dsRNA knockdown as a positive control, and *mod(mdg4)67.2* knockdown as a control for knockdown efficiency in the screen. Briefly, 0.25 µg of dsRNAs that target *mcherry*, *su(Hw),* or *mod(mdg4)67.2* was pre-spotted before dispensing modified Kc cells into 384-well plates. Cells were soaked with dsRNA in a 25 °C incubator for 4 d and then cold shocked at 4 °C for 2.5 h before fixation with 8% PFA for 10 min. After washing twice in PBS and staining with DAPI (2 μg/mL) for 10 min, cells were sealed with Alumaseal and stored at 4 °C. Images were taken on a high-throughput microscope (CellVoyager CV7000 (Yokogawa) spinning-disk confocal) and processed using Columbus software (PerkinElmer). Before applying machine learning in Columbus, parameters for large foci were determined manually. Data analysis from two biological replicates of the screen was conducted using the cellHTS2 R package [49] (V2.44.0) to determine the *Z*′ and *Z* score, which are statistical measures used in high-throughput screening to optimize parameters and identify candidates [50]. By calculating the standard deviation from the mean of the control data, *Z*′ shows the discrepancy in positive and negative controls to determine overall assay quality. A *Z*′ score of > 0.5 indicates a good, reliable assay, and 1.0 indicates an ideal assay. *Z* scores of each RNAi treatment in the library were calculated by ranking based on their phenotypic effect measured as the number of large insulator bodies and total imaged cells in each well (Additional File 2: Table S1–2). For all follow-up analyses, cells were imaged on glass slides, and this experimental condition leads to an overall increase in focus number relative to 384-well plates, but trends after knockdowns remain consistent [13].

### Antibodies

For western blotting, rabbit anti-CP190 [51] (laboratory-made; 1:10,000), guinea pig anti-Su(Hw) [52] (laboratory-made; 1:1000), rabbit anti-Mod(mdg4)67.2 [53] (laboratory-made; 1:1000), mouse anti-α-Tubulin (Sigma T6074; 1:50,000), mouse anti-Eff (human UBE2D2; Santa Cruz Biotechnology (SCBT) sc-100617; 1:1000), mouse anti-Cul4 (human Cul4; SCBT sc-377188; 1:1000), rabbit anti-Cul4 (human Cul4; Novus NB100-2267, 1:1000), mouse anti-Lamin B (DSHB ADL67.2; 1:10,000), and rabbit anti-Polycomb (gift from Cavalli lab; 1:4,000) [54] were used.

For immunostaining, rabbit anti-CP190 [22] (laboratory made; 1:2000 for IF, 1:1000 for IF and FISH) and guinea pig anti-Su(Hw) [52] (laboratory made; 1:1000) were used. Secondary goat antibodies labeled with AlexaFluor 488, AlexaFluor 546, or AlexaFluor 647 (Molecular Probes) were used at 1∶1000.

For chromatin immunoprecipitation (ChIP), rabbit anti-Cul4 (human Cul4; Novus NB100-2267), mouse anti-Cul4 (human Cul4; SCBT sc-377188), mouse anti-Eff (human UBE2D2; SCBT sc-100617), rabbit anti-Eff (Novus, NBP2-20,783), rabbit anti-CP190 (laboratory-made) [51], and guinea pig anti-Su(Hw) [52] were used. The alignment of Cul4, Eff, and the antigen region of the human ortholog used for developing antibodies is detailed in Additional File 1: Fig. S4.

### Luciferase insulator barrier activity assay

Using the Bright-Glo™ Luciferase Assay System, the luciferase insulator barrier activity assay was carried out as described previously (Promega) [13]. Signals were captured using a Spectramax II Gemini EM plate reader (Molecular Devices). Luciferase levels were quantified in a single panel for all genotypes, containing twelve individual male third-instar larvae. For each larva, luciferase values were normalized to the total protein determined by BCA reagent (Thermo Scientific) to obtain relative luciferase activity. Data were arranged into a box-and-whisker plot and analyzed with one-way ANOVA followed by a Tukey HSD post hoc test to obtain *p*-values for each pairwise comparison.

### ChIP quantitative PCR

We collected second instar larvae harboring the *gypsy* insulated UAS-luciferase reporter, using *da*-gal4 to drive *eff*^*RNAi*^ or *cul4*^*RNAi*^ and performed quantitative PCR after ChIP with anti-Histone H3 (Abcam, ab1791), CP190, or Su(Hw). ChIP DNA samples were amplified using site-specific primer sets (Additional File 2: Table S8) and quantified using SYBR Green (Applied Biosystems) calibrated against input chromatin. Experiments were performed with two independent biological replicates, and each sample was quantified using two technical replicates (Additional File 2: Table S7).

### Co-immunoprecipitation

Embryonic nuclear extract from mixed-stage (0–24 h) embryos was prepared as described previously [21]. Nuclei were lysed with HBSMT nuclear lysis buffer (50 mM HEPES, 1 M KCl, 150 mM NaCl, 3 mM MgCl_2_, 0.3% Triton X-100 at pH 7, 1 mM PMSF, and Roche cOmplete protease inhibitor), sonicated 10 times with 10 s on and 10 s off, and then centrifuged to collect the soluble fraction of the extracts. To prepare for immunoprecipitation (IP), 25 µL of Protein A Sepharose beads (GE Healthcare) were washed three times with nuclear lysis buffer. 10 µL of rabbit anti-Eff (Novus, NBP2-20,783), 10 µL of rabbit anti-Cul4 (Novus, NB100-2267), 10 µL of rabbit IgG (Santa Cruz), 3 µL of anti-serum against CP190 (rabbit, laboratory-made), and 3 µL of rabbit normal serum (Covance Research Product) were incubated with Sepharose beads for 1 h at 4 °C, and the unbound portion was removed by centrifugation. 0.2 M of sodium borate at pH 9 was used to wash beads three times before crosslinking antibodies to beads with 20 mM Dimethyl pimelimidate in sodium borate for 30 min at RT. After centrifugation, beads were quenched with ethanolamine for 2 h and washed three times with lysis buffer. After crosslinking, 500 µg of nuclear extract was added to each immunoprecipitation reaction and incubated overnight at 4 °C. Beads were collected by centrifugation the next day and washed three times with nuclear lysis buffer. Samples were eluted with SDS sample buffer by boiling, separated using SDS-PAGE, transferred to nitrocellulose membrane in 10 mM CAPS, pH 11, and detected by western blotting.

### Immunofluorescence and fluorescence in situ hybridization with Oligopaints

*GypsyF* and *gypsyR* probes to Chr3L were designed in a previous study, and IF combined with FISH followed the same procedure as previously reported [13]. Briefly, FISH libraries for Chr3L were designed using the Oligominer pipeline based on the dm6 reference genome, and two libraries were multiplexed with barcodes: (i) *gypsy* forward probe (*gypsyF*), (ii) *gypsy* reverse control probe (*gypsyR*). G*ypsyF* sub-libraries contain sites with Su(Hw), Mod(mdg4)67.2, and CP190 ChIP-seq colocalization in Kc cells. For the reverse control sub-libraries, the chromosomal coordinates of *gypsy*F probes were reversed in one dimension, and oligos in the control sets that overlapped with *gypsyF* libraries were excluded. Therefore, random control sets contain only chromatin devoid of CP190, Su(Hw) and Mod(mdg4)67.2 three-way binding. Slides were washed twice in PBST, once in 2 × SSCT (0.3 M NaCl, 0.03 M sodium citrate, 0.1% Tween-20) and once in 2 × SSCT/50% formamide at RT for 5 min. Slides were pre-denatured at 92 °C in 2 × SSCT/50% formamide for 2.5 min, then at 60 °C in 2 × SSCT/50% formamide for 20 min. After that, 100 pmol of primary Oligopaint probe was mixed in hybridization buffer (10% dextran sulfate/2xSSCT/50% formamide/4% polyvinylsulfonic acid (PVSA)) with a final volume of 25 μL and dispensed onto slides. Slides were covered with a coverslip, sealed with rubber cement, denatured at 92 °C for 2.5 min and transferred to a 37 °C humidified chamber overnight. The next day, slides were washed in 2 × SSCT for 15 min at 60 °C, 15 min at RT, and 5 min at RT in 0.2 × SSC. After adding secondary fluorophore probes in hybridization buffer (10 pmol/25 μL), slides were covered, sealed and incubated in a 37 °C humidified chamber for 2 h before being washed in 2 × SSCT for 15 min at 60 °C and RT sequentially, and then in 0.2 × SSC at RT for 5 min. Slides were stained with DAPI (2 μg/mL, Sigma) for 10 min and mounted in Prolong Diamond (Life Technologies).

### Imaging, quantification, and data analysis

Images were captured using a Leica DMi 6000B widefield fluorescence microscope using Leica DFC9000 sCMOS Monochrome Camera and a 1.4 NA 63 × objective. For quantification of Mod(mdg4)67.2-GFP foci in immunostaining, we used the TANGO 3D-segmentation plugin for Fiji [55] to determine large GFP foci (> 0.5 μm^3^) [13].

Huygens Professional software (Scientific Volume Imaging, Hilversum, Netherlands) was used to deconvolve IF/FISH images. After deconvolution, images were imported, segmented, and analyzed using TANGO. *GypsyF* and *R* paints on Chr3L were segmented using the “Hysteresis” algorithms. The average radial position of paints in the nucleus was determined by quantitative shell analysis, with five shells of equal volume to quantify the percentage of chromosome signals in each concentric shell, in which shell 1 is the closest to the nuclear periphery and shell 5 is the nuclear center. We summarized data from three biological replicates, and each dot represents the average of > 500 cells from one replicate. Statistical tests were performed using Prism 10 software by GraphPad. Tiff files for example images were maximal projections of 36 *Z*-stacks created in ImageJ.

### ChIP and ChIP-seq library preparation

Two to 3 × 10^7^ cells were fixed by adding 1% formaldehyde directly to cells in culture medium for 10 min at RT with gentle agitation. 0.125 M glycine was used to quench formaldehyde with gentle agitation for 5 min. Cells were centrifuged at 500 × *g* for 5 min and washed twice with ice-cold PBS. Chromatin preparation and chromatin immunoprecipitation (ChIP) were performed following earlier described methods [56]. Libraries were prepared according to the manufacturer’s protocol (NEB; Ultra II for DNA Library Prep Kit; E7645). All samples were sequenced with Novaseq 6000 (Illumina) using 50 bp single-end sequencing at the NHLBI DNA Sequencing and Genomics Core Facility.

### ChIP-seq data analysis

FASTQ files of sequenced single-end 50-bp reads were trimmed using cutadapt v3.4 [57] with arguments “-a AGATCGGAAGAGCACACGTCTGAACTCCAGTCA”, “–nextseq-trim 20”, “–minimum-length 25”, and “–overlap 6”. Bowtie2 v2.4.2 [58] with default arguments was used to map the trimmed reads to the FlyBase r6-35 genome assembly. Then, multimapping reads were removed using the samtools v1.12 [59] view command with the argument -q 20. Picard MarkDuplicates v2.25.2 (http://broadinstitute.github.io/picard/index.html) was applied to remove duplicates from mapped, uniquely-mapping reads. Peaks were called by MACS2 v2.2.7.1 [60] (https://github.com/taoliu/MACS) by providing replicate IPs and inputs as multiple BAMs with arguments “-f BAM”, “–gsize = dm”, “-q 0.000000001”, and “–mfold 5 50”. Source code originated from GitHub (https://github.com/lcdb/lcdb-wf/tree/master/workflows/chipseq). The resulting peak files were imported into R as GRanges objects, and reproducible peaks were identified by selecting genomic intervals shared across all three replicates using the findOverlaps function. These reproducible regions were exported as BED files and used as the merged peak sets for each factor. To obtain high-confidence peak sets, merged peaks were filtered against the dm6 ENCODE blacklist using bedtools intersect -v, which removes any interval that overlaps a blacklist region. The filtered peaks were then sorted and deduplicated to eliminate redundant entries. The final BED files generated from this process were used for all downstream analyses.

FlyBase release 6.35 annotations were used to annotate peaks as previously described [13]. Promoters were defined as each transcript’s transcription start site (TSS) plus 1500 bp upstream. Using the gffutils (https://github.com/daler/gffutils) method FeatureDB.create_introns, introns were designated as the space between exons in a per-transcript manner. Intergenic regions were from all regions between gene bodies. Overlapping peaks were determined using pybedtools BedTool.intersect with the v = True or u = True arguments, respectively. Peaks in each set were intersected with the annotations using this hierarchy: “promoter > exon > intron > intergenic”, and the peak was classified according to the highest priority feature. For example, a peak simultaneously intersecting a promoter of one isoform and an intron of a different isoform would be prioritized as “promoter”. To quantify the percentages across annotated peaks of different types of peaks (all CP190, all Cul4, shared CP190 and Cul4 peaks, all Su(Hw)), the number of peaks in each class was divided by the total number of peaks of that type.

Binary heatmaps were generated using pybedtools v 0.8.2 [61, 62] as previously described [13]. Heat maps were generated using deeptools (v3.5.1). ChIP reads were normalized to reads of input samples and mapped in a 1.5-kb window centered on the CP190, Su(Hw) or Cul4 summit and sorted by descending signal in control cells.

### Differential ChIP-seq

Diffbind v3.10.0 using R 4.3.0 [63] was used to determine differential peaks between control and knockdown cells. For each replicate, multimappers and duplicates were removed from IP and input BAM files as described above, and input files consisted of the final peak calls as mentioned above. We used the config object “data.frame(RunParallel = FALSE, DataType = DBA_DATA_FRAME, AnalysisMethod = DBA_EDGER, bCorPlot = FALSE, bUsePval = FALSE, minQCth = 15, fragmentSize = 0)” and other default settings. Data frames were exported with the dba.report function with parameters “th = 1, normalize = DBA_NORM_TMM”. Finally, differentially lost or gained peaks were those that had a log2 fold change of < 0 or > 0, respectively, and FDR < 0.05.

### Nascent EU-RNA labeling and library preparation

Kc cells were incubated with 0.2 mM 5-Ethynyl uridine (EU) for 1 h, and RNA was extracted with the RNeasy Plus Mini Kit (Qiagen). Samples for neuRNA-seq were labeled and captured using the Click-iT Nascent RNA Capture Kit (Thermo Fisher Scientific) according to the manufacturer’s protocol. Biotinylation of RNA by the Click reaction was performed with 0.5 mM biotin azide using 1 µg of EU-RNA, and then the biotinylated RNA was captured with 50 μL of Dynabeads Streptavidin T1 magnetic beads. The neuRNA-seq libraries were prepared using Universal Plus Total RNA-Seq with *Drosophila* AnyDeplete (Tecan Genomics) and sequenced on Novaseq 6000 using paired-end sequencing at the NHLBI DNA Sequencing and Genomics Core Facility.

### neuRNA-seq data processing

Low-quality bases and adaptors were trimmed from sequence reads using cutadapt v3.4 with parameters “–q 20 -minimum-length 25 -a AGATCGGAAGAGCACACGTCTGAACTCCAGTCA -A AGATCGGAAGAGCGTCGTGTAGGGAAAGAGTGT.” The resulting reads were mapped to the FlyBase r6-35 reference genome using HISAT2 v2.2.1 [64] with default parameters. Aligned reads were counted using subread featureCounts v2.0.1 [65] with optimized parameters. The “-t gene” option was used to quantify the reads on the gene feature for neuRNA-seq, and “-s1” option was used specifically for this sense-stranded library. Count tables were used as input files for the downstream differential analysis.

### Differential expression analysis in neuRNA-seq

Count tables from independent neuRNA-seq experiments were independently imported into DESeq2 v1.40.1 [66] using R4.3.0 and normalized by design “ ~ treatment” and with otherwise default parameters. DEGs were those with FDR < 0.05.

### Distance analysis of promoter to the closest TAD border

TAD borders were downloaded [12] and lifted over to the dm6 assembly (liftover, https://genome.ucsc.edu/cgi-bin/hgLiftOver). The distance of the promoter to the TAD border was calculated using pybedtools v0.8.2 with closest operations to sort and compute the minimum distance to the TAD border. Seaborn v0.11.1 (seaborn.kdeplot) was used to calculate the kernel density of distance for up-regulated, down-regulated and unchanged genes. Based on the kernel density estimation, cumulative distribution plots were generated. Matplotlib v3.4.1 package was used to generate histograms of the density of gene distance to the nearest TAD border (matplotlib.pyplot.hist, bin = 100).

### Fisher’s exact tests

We use two-sided FET to calculate the *P*-value and the odds ratio. In Fig. 3F, FET examines the frequency of Cul4 sites localized at TAD borders that overlap with binding of CP190. For Figs. 4C, D, and Additional File 1: Fig. S5, these tests were used to examine whether DEGs show correlation with binding of CP190, Cul4 or Su(Hw) at promoters compared with unaffected genes after depletion of Eff or Cul4. For Fig. 5C, to examine whether CP190 chromatin binding is enriched with Cul4 binding, FET was used to calculate CP190 peaks that are changed/not changed in Cul4- and Eff-depleted cells versus overlapping/not overlapping with Cul4 binding in control conditions. Flybase r6-35 gene annotation gtf file was used to define TSS, and a gene promoter region was selected as ± 1 kb of the TSS to intersect with ChIP peaks. Moreover, promoter regions were merged if there was any overlap. FET was performed using the number of gene promoters occupied/not occupied by feature peaks from the group of down-regulated (log2fold < 0, *P*_adj_ < 0.05), up-regulated (log2fold > 0, *P*_adj_ < 0.05) and unchanged gene promoters, respectively analyzed using DESeq2 and implemented by scipy v1.6.2 (scipy.stats.fisher_exact) in Python v3.9.4.

### Motif analysis

To search de novo motifs bound by Cul4 or CP190, we used the HOMER [67] (v4.11.1) findMotifsGenome.pl script with default settings. The highest-scoring sequences with percentages in targets and *p*-values are presented.

## Supplementary Information

Additional file 1. Fig. S1 - Localization of *gypsy* insulator bodies is disrupted in knockdown cells. Fig. S2 - Eff, Cul4 and Topors do not affect *gypsy *insulator protein levels. Fig. S3 - ChIP-qPCR in *gypsy* insulated UAS-luciferase transgenic larvae. Fig. S4 - Validation of anti-human Cul4 antibodies for ChIP-seq in *Drosophila*. Fig. S5 - CP190 and Cul4 are highly enriched at DEG promoters after depletion of Eff or Cul4. Fig. S6 - Differential CP190 peaks after either Eff or Cul4 depletion are repositioned. Fig. S7 - Depletion of Eff affects the 3D organization of *gypsy* insulator binding sites.

Additional file 2. Tables S1-S3. Z score of GFP foci number, cell number, and GFP intensity for ubiquitin pathway-related genes. Tables S4-5. Mass spectrometry results after pulling down CP190 and Su(Hw) from *Drosophila* embryonic nuclear extract. Table S6. Luciferase assay p-values for knockdowns and controls. Table S7. Percent of input DNA precipitated by ChIP-qPCR. Table S8. List of primers for dsRNA and ChIP-qPCR.

Additional file 3. Original western blot images.

## Data Availability

The accession numbers for the FASTQ raw data, processed data such as BigWig files and peak files for all sequencing samples generated in this study are deposited in NCBI GEO, Bioproject Accession: GSE243451 (https://identifiers.org/geo:GSE243451) [68] and GSE243452 (https://identifiers.org/geo:GSE243452) [69]. Kc167 TAD borders were obtained from https://github.com/deeptools/HiCBrowser, which were analyzed in Ramirez et al. [12] (GEO: GSE97965 (https://identifiers.org/geo:GSE97965)). Data were lifted over to the dm6 assembly.

## Abbreviations

3D: Three-dimensional
ChIP: Chromatin immunoprecipitation
CP190: Centrosomal protein 190
CRL: Cullin-RING E3 ubiquitin ligase
Cul4: Cullin 4
DEG: Differentially expressed genes
DRSC: Drosophila RNAi Screening Center
Eff: Effete
FDR: False discovery rate
FET: Fisher’s exact test
IP: Immunoprecipitation
Kc cells: Kc167 hemocyte cells
Mod(mdg4)67.2: Modifier of mdg4 67.2 kDa isoform
NE: Nuclear extract
neuRNA-seq: Nascent EU RNA sequencing
Su(Hw): Suppressor of Hairy wing
SUMO: Small ubiquitin-like modifier
TAD: Topologically associating domain
Topors: Topoisomerase I-interacting RS protein
TSS: Transcription start site
UAS: Upstream activating sequence
