# Supplementary material for: Effete and Cullin 4 affect nuclear organization of the gypsy chromatin insulator
Source: BMC Biol. 2026 Apr 17;24:126. doi: 10.1186/s12915-026-02596-6 (PMC13217776; doi:10.1186/s12915-026-02596-6)
Supplement: Supplementary file 1 — Additional file 1. Fig. S1 - Localization of gypsy insulator bodies is disrupted in knockdown cells. Fig. S2 - Eff, Cul4 and Topors do not affect gypsy insulator protein levels. Fig. S3 - ChIP-qPCR in gypsy insulated UAS-luciferase transgenic larvae. Fig. S4 - Validation of anti-human Cul4 antibodies for ChIP-seq in Drosophila. Fig. S5 - CP190 and Cul4 are highly enriched at DEG promoters after depletion of Eff or Cul4. Fig. S6 - Differential CP190 peaks after either Eff or Cul4 depletion are repositioned. Fig. S7 - Depletion of Eff affects the 3D organization of gypsy insulator binding sites. [file 12915_2026_2596_MOESM1_ESM.docx]

**
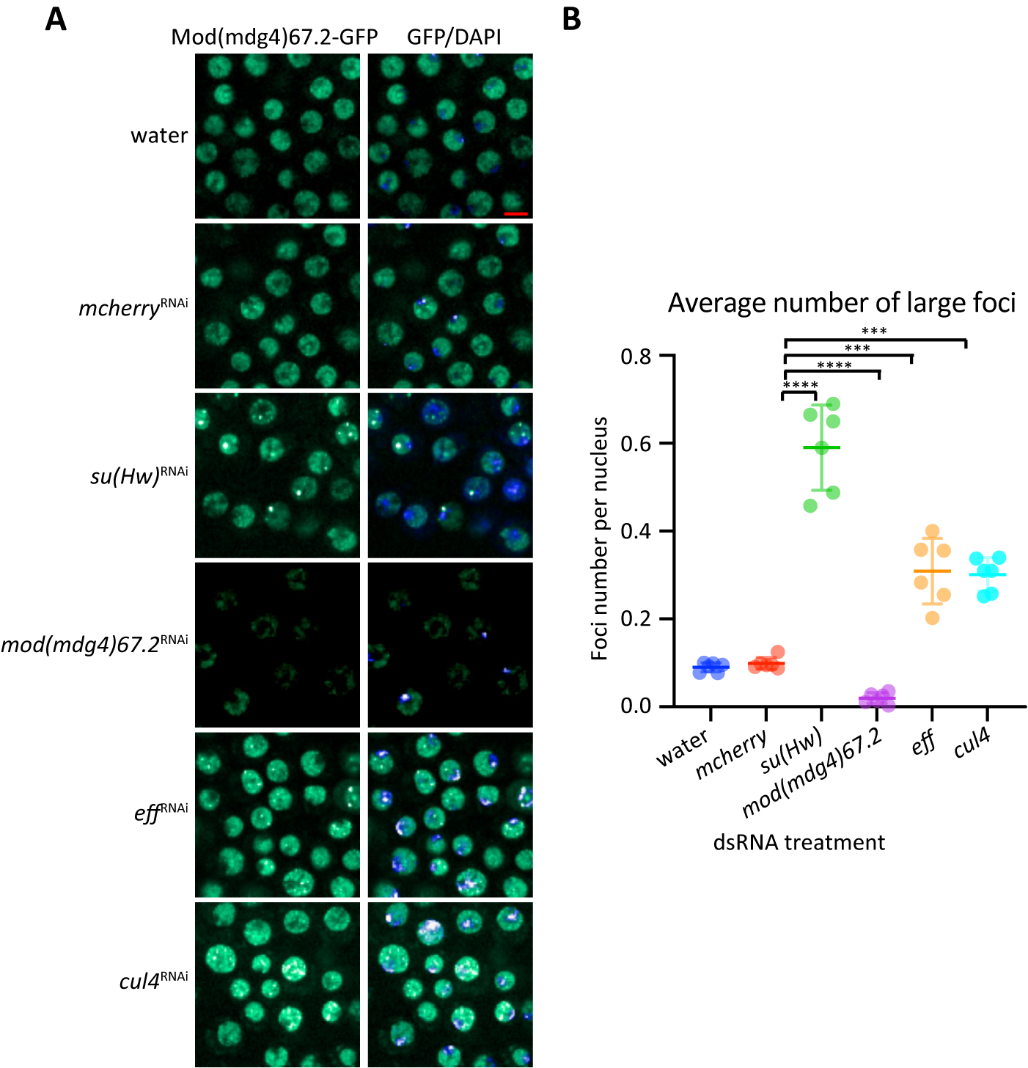
**

**Fig. S1 Localization of *gypsy* insulator bodies is disrupted after depletion of Su(Hw), Eff, or Cul4.** Representative images of Mod(mdg4)67.2-GFP signal in water, *mcherry, su(Hw), mod(mdg4)67.2, eff*, and *cul4* dsRNA-treated cells in 384-well plates using an automated microscope for screening. In control cells, Mod(mdg4)67.2-GFP signal is diffuse in the nucleus. GFP foci/insulator bodies are highly visible in Su(Hw), Eff, and Cul4 depleted cells and can be easily detected using semi-automatic Columbus imaging software. Oversaturated DAPI signal is indicated in white in the right panel. Scale bar: 5 μm. (B) Quantification of average number of large (volume > 0.5 μm3) GFP foci after each indicated knockdown. Each dot in the plot represents one well replicate, and at least 6 wells from both screening runs are shown, with > 1000 cells quantified in each replicate. Data were analyzed using a Paired t-test, and error bars represent standard deviation (s.d.). ****P* < 0.001, *****P* < 0.0001.

**
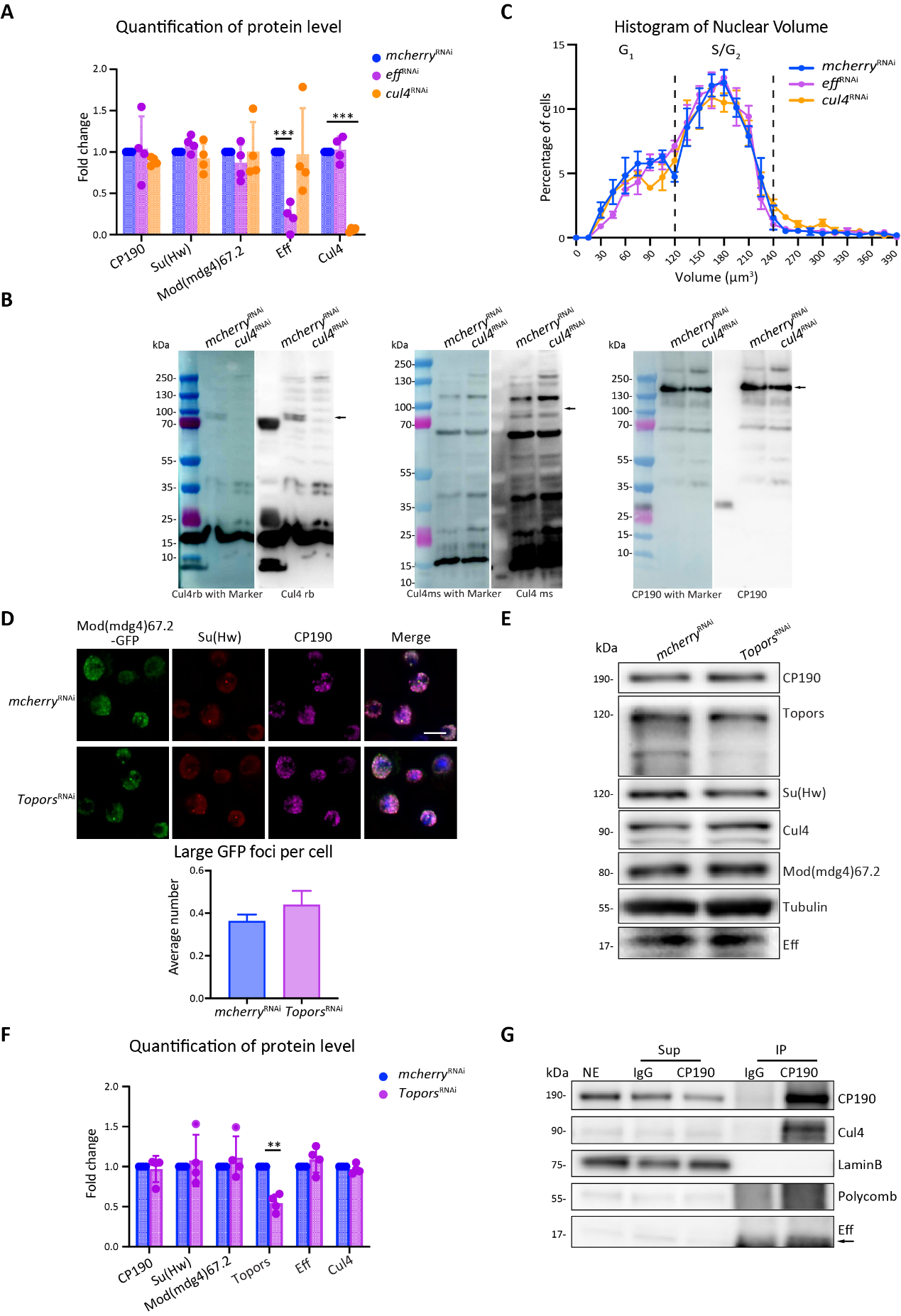
Fig. S2 Eff, Cul4, and Topors do not affect *gypsy* insulator protein levels, and Topors does not significantly impact the localization of Mod(mdg4)67.2-GFP.** (A) Quantification by Western blotting of CP190, Su(Hw), Mod(mdg4)67.2, Eff, and Cul4 protein levels relative to Tubulin loading control and normalized to the *mcherry* RNAi control in Kc cells treated with indicated dsRNA. Data are from 4 replicates. (B) Full membrane Western blots with rabbit anti-Cul4, mouse anti-Cul4, and anti-CP190 in control and *cul4* knockdown Kc cells. Visible image of pre-stained marker is overlaid with chemiluminescent signal of anti-Cul4 (rabbit) on left paired with anti-Cul4 signal alone. Samples were stained in parallel lanes with anti-Cul4 (mouse) or anti-CP190 and imaged similarly. Although multiple bands are detected, specific reduction of the band corresponding to full-length Cul4 confirmed the specificity of the antibodies. (C) Percentage of cells in each cell cycle stage after knockdown of *eff* or *cul4* based on nuclear volume. Data is represented as the mean ± SE of the average of three biological replicates. (D) Representative images of Mod(mdg4)67.2-GFP after growth at 25˚C and depletion of Topors on glass slides. No significant change in number was detected for large GFP foci, and data were collected from three replicates. (E) Western blotting in Kc control and *Topors* knockdown cells showing knockdown efficiency and no effect on protein levels of *gypsy* insulator proteins, Eff, or Cul4. (F) Quantification by Western blotting of CP190, Su(Hw), Mod(mdg4)67.2, Topors, Eff, and Cul4 protein levels in Kc cells treated with *Topors* dsRNA. (G) Immunoprecipitation of Eff and Cul4 using normal IgG or anti-CP190 is shown. Polycomb is shown as a negative control for interaction, and the Eff band in CP190 IP is indicated with an arrow.


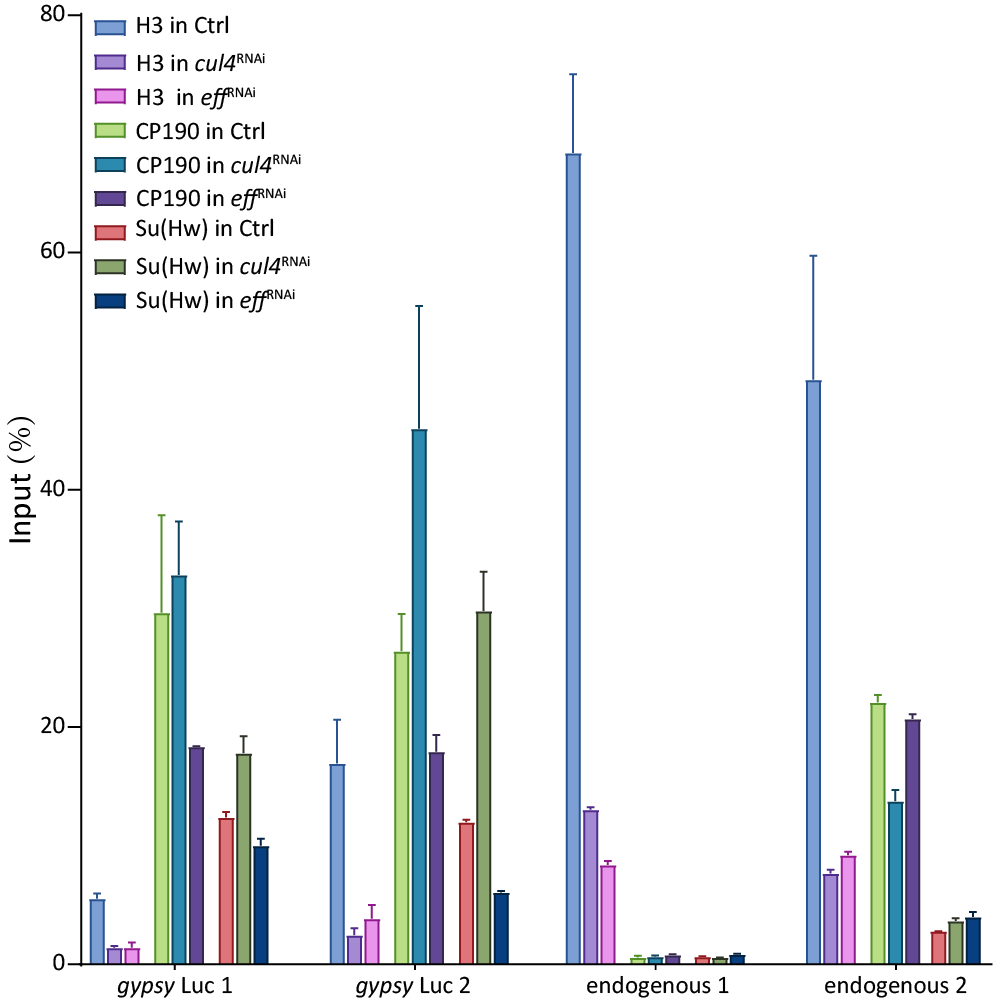


**Fig. S3 ChIP-qPCR of Histone H3, CP190 and Su(Hw) in *gypsy* insulated UAS-luciferase transgenic larvae.** Percent of input DNA precipitated for each ChIP sample is shown for control control versus *cul4^RNAi^* or *eff^RNAi^* larvae driven with *da-Gal4*. We performed the entire experiment twice using two technical replicates for PCR measurement, and we displayed the data for the mean ± SE of one set of biological replicates. Data of two biological replicates are provided in Additional file 2: Table S7.

**
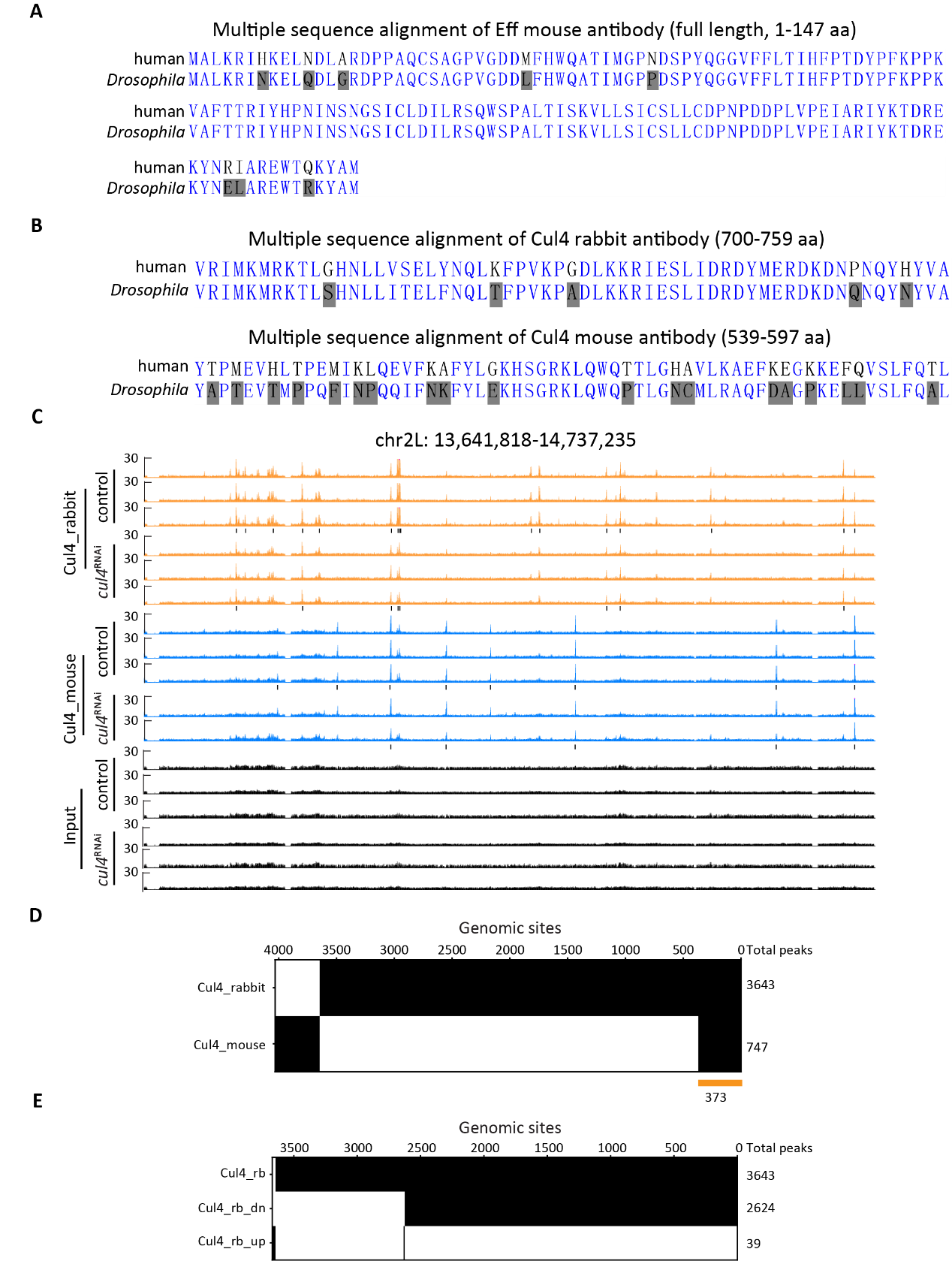
**

**Fig. S4 Validation of anti-human Cul4 antibodies for ChIP-seq in *Drosophila*.** (A-B) Alignment of antigen regions for human Eff and Cul4 with respective homolog in *Drosophila*. Conserved amino acids are indicated in blue and non-conserved are shown in gray boxes. (C) ChIP for Cul4 using two independent antibodies from different sources (rabbit and mouse). Black bars show peaks called across three biological replicates. Merged peaks using rabbit antibody is 3643, and there are 747 using mouse antibody. After depletion of Cul4, the number of peaks called for rabbit is 2090, and 502 are called for mouse. Note that only two biological replicates are used for mouse anti-Cul4 after Cul4 depletion. (D) Binary heatmap of peaks using rabbit or mouse Cul4 antibodies. The total number of peaks is shown on the right, and the yellow bar indicates overlapping sites between Cul4 antibodies. (E) Binary heatmap of ChIP-seq peaks of rabbit anti-Cul4 of control peaks versus differentially decreased and increased peaks in the Cul4 knockdown condition. Rabbit anti-Cul4 profiles were used for downstream analyses due to higher conservation of the antigen.


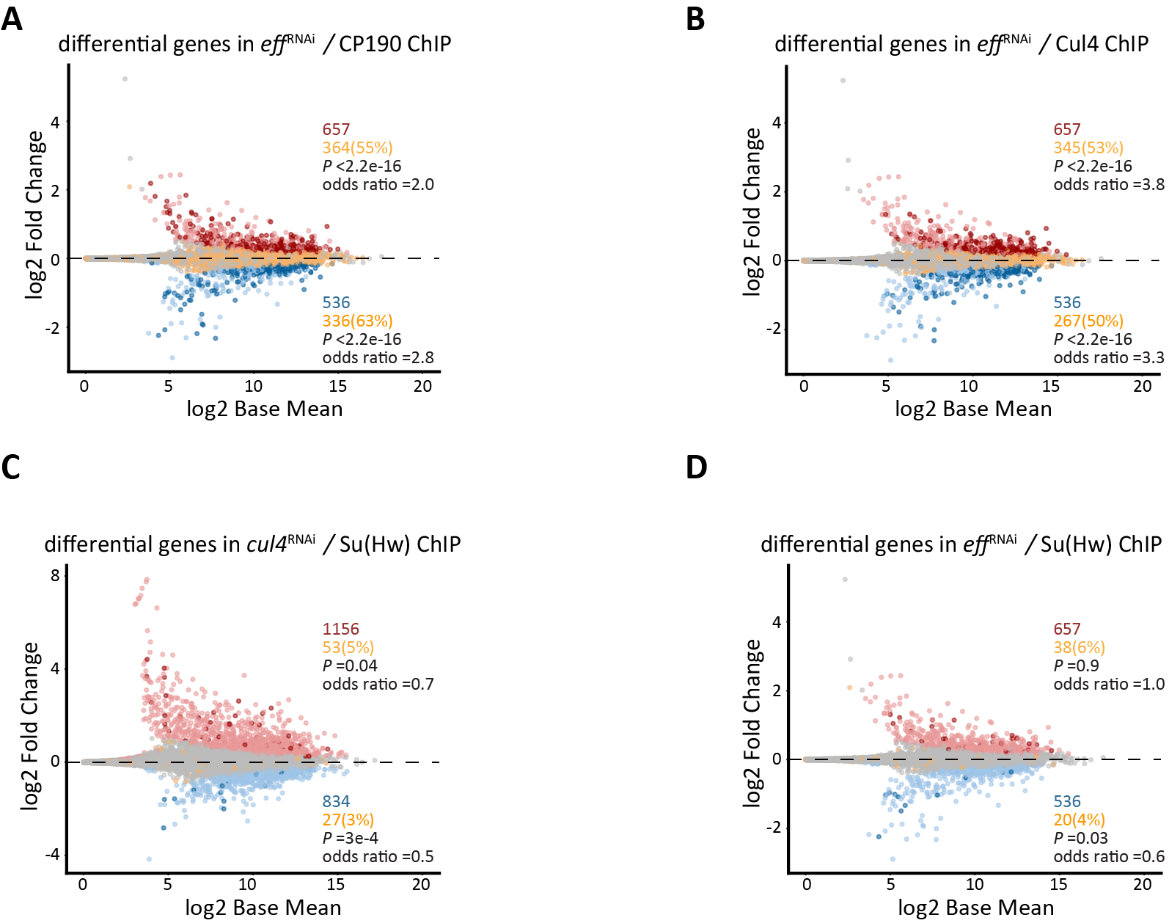


**Fig. S5 CP190 and Cul4 but not Su(Hw) are highly enriched at DEG promoters after depletion of Eff or Cul4.** (A-B) MA plots showing occupancy of CP190 and Cul4 in affected genes of neuRNA-seq in *eff* knockdown cells. (C-D) MA plots indicating the correlation of neuRNA-seq affected genes after depletion of *cul4* (C) or *Eff* (D) with binding of Su(Hw) at their promoters. Statistically significant changes include up- (red) and down-regulated (blue) genes using *P*_adj_ < 0.05. Unchanged genes without protein binding are indicated in gray. Gene promoters of unchanged genes containing CP190, Cul4 or Su(Hw) peaks are colored in orange. Up-regulated genes with (dark red) or without (light red) peak at the promoter, and down-regulated genes with (dark blue) or without (light blue) binding at the promoter are shown. Two-sided Fisher’s exact test was used to determine the *P*-value and odds ratio when comparing the binding frequency at the promoter of affected genes with unchanged genes.


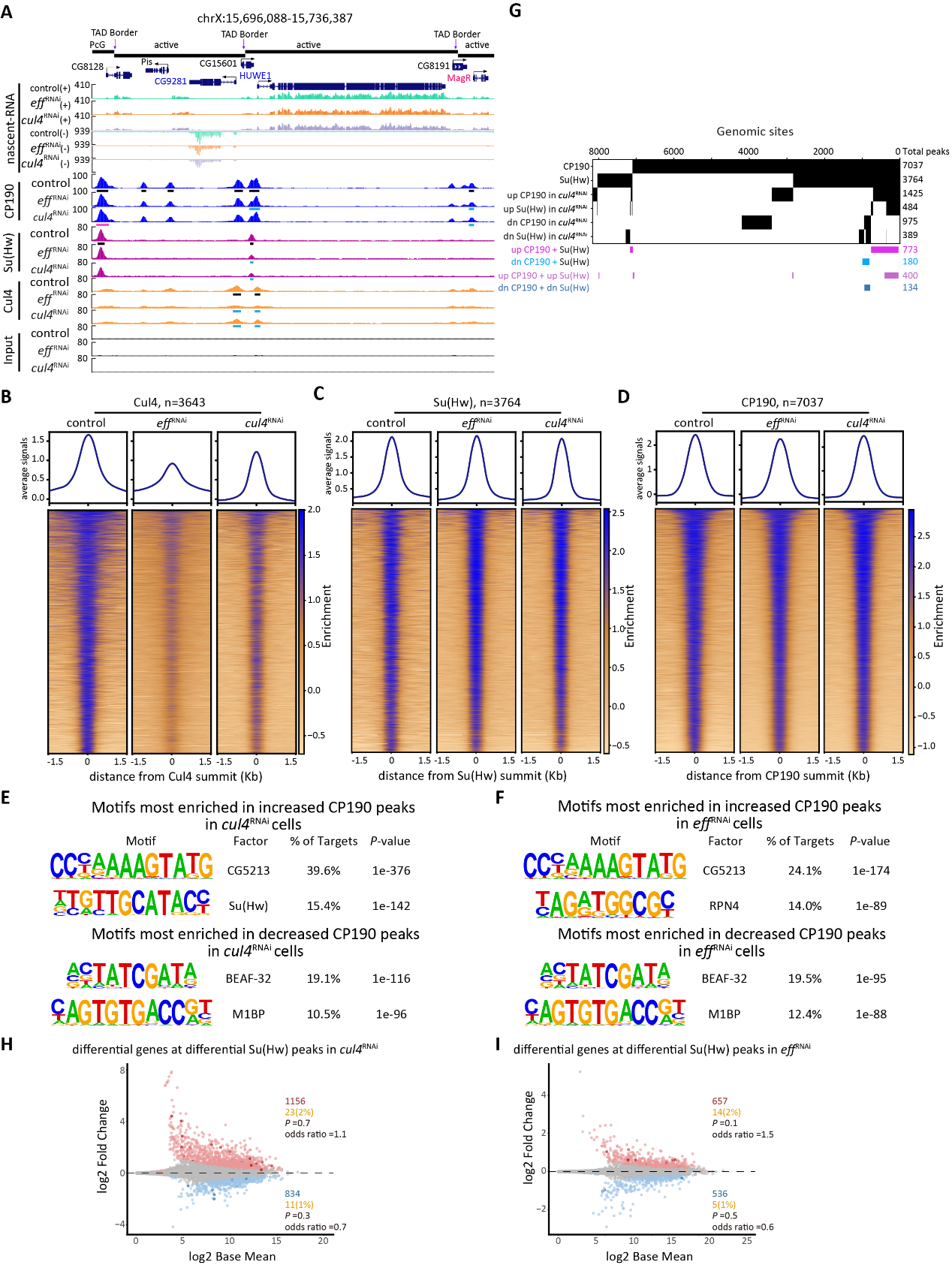
**Fig. S6 Differential CP190 peaks after either Eff or Cul4 depletion overlap considerably and are repositioned.** (A) Representative example of ChIP and neuRNA-seq tracks in control, *eff* and *cul4* RNAi cells. Decreased and increased peaks/genes are indicated in blue and pink, respectively, in knockdown conditions. (B) ChIP-seq signal at called peaks centered on their summit of Cul4 (B), Su(Hw) (C) and CP190 (D) in dsRNA transfected cells as indicated, which are sorted by descending signal in control cells. Cul4 signals are greatly diminished after depletion of Eff or Cul4. (E-F) Motif enrichment of differential CP190 peaks after depletion of Cul4 (E) or Eff (F). (G) Binary heatmap of Cul4, CP190, Su(Hw), and differential binding sites of CP190 and Su(Hw) in cells depleted of Cul4. 773 (54% of 1425) increased CP190 and 180 (18% of 975) decreased CP190 after depletion of Cul4 overlap with Su(Hw) in the control condition. Dn denotes decreased peaks, and up indicates increased. Rectangles indicate overlapping sites of differential peaks of CP190 and Su(Hw) or matching differential Su(Hw), respectively. (H-I) MA plots showing the redistribution of Su(Hw) at differentially expressed genes of neuRNA-seq in *cul4* (H) and *eff* (I) knockdown cells.

**
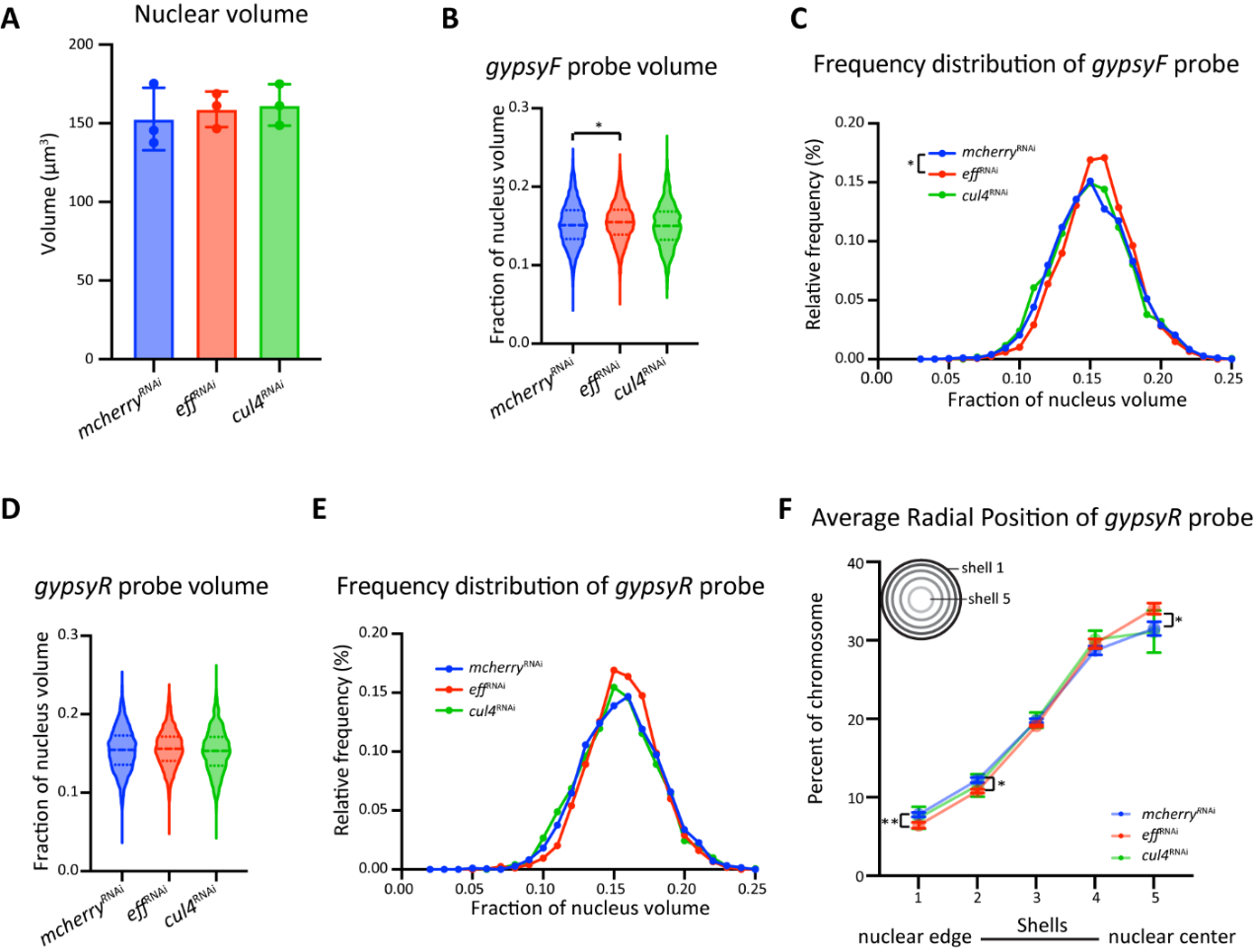
**

**Fig. S7 Depletion of Eff but not Cul4 affects the 3D organization of *gypsy* insulator binding sites.** (A) Nuclear volume does not change after any dsRNA knockdown. Data are from three biological replicates, with n > 500 cells per replicate. (B-E) Quantification of probe volume relative to nuclear volume in a single replicate. The volume of *gypsyF* paint (B) is significantly increased in *eff* knockdown cells but not in *cul4* knockdown cells. No change in *gypsyR* paint volume (D) was detected in any treatment. Frequency distribution of *gypsyF* (C) and *gypsyR* (E) paint volume in cell population. Student’s t-test was applied, and error bars show standard deviation, * is *P* < 0.05. (F) Shell analysis to determine radial position of *gypsyR* paint in the nucleus. The nucleus was divided into five shells of equal volume. Shell 1 is the closest to the periphery, and shell 5 is the center. n > 500 cells. Paired-t test was used to obtain statistically significant difference from the median of three biological replicates. * *P* < 0.05, ***P* < 0.01, ****P* < 0.001, and error bars show standard deviation.
