# Supplementary material for: Effete and Cullin 4 affect nuclear organization of the gypsy chromatin insulator
Source: BMC Biol. 2026 Apr 17;24:126. doi: 10.1186/s12915-026-02596-6 (PMC13217776; doi:10.1186/s12915-026-02596-6)

**Original uncropped Western blot images**

**Corresponding to Figure 1C**

Sample lanes (left to right): Marker, *mcherry^RNAi^, cp190^RNAi^, su(Hw)^RNAi^, mod(mdg4)67.2^RNAi^, mcherry^RNAi^, eff^RNAi^, mcherry^RNAi^, cul4^RNAi^*, WT1, WT2, Marker

Note that chemiluminescent signal is shown on left, and overlay of chemiluminescence and visual light is shown on right.

α-CP190


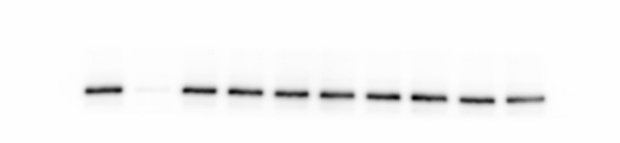

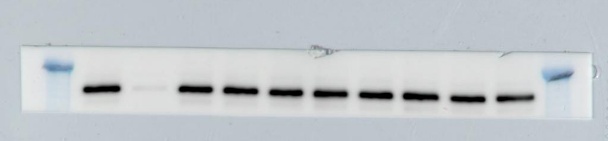


α-Su(Hw)


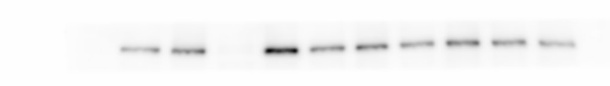

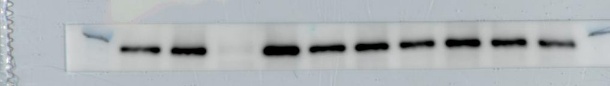


α-Mod(mdg4)67.2


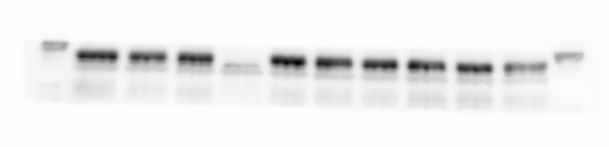

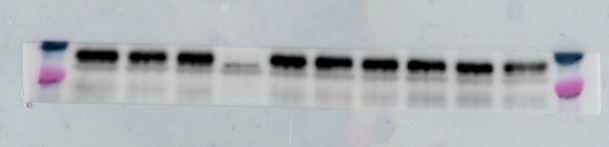


α-Eff (mouse)


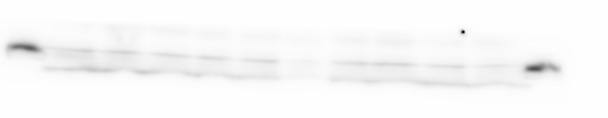

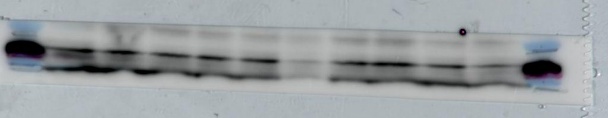


α-Tubulin


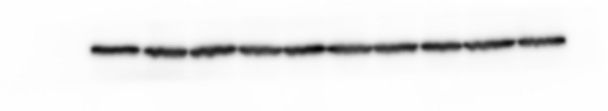

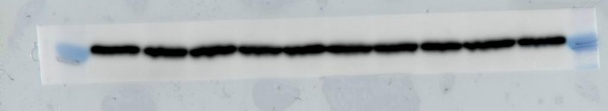


Sample lanes (left to right): Marker, *mcherry^RNAi^, cp190^RNAi^, su(Hw)^RNAi^, mod(mdg4)67.2^RNAi^,* WT1, WT2, *mcherry^RNAi^, eff^RNAi^, mcherry^RNAi^, cul4^RNAi^*, Marker. Note that these are the exact same samples as loaded in the top set.

α-Cul4 (mouse)


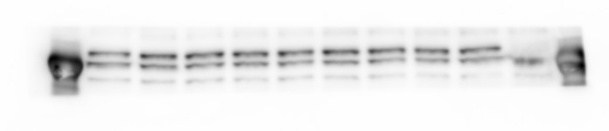

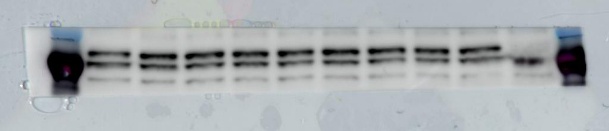


**Corresponding to Figure 1D**

Sample lanes correspond to labeling in main figure.

α-CP190


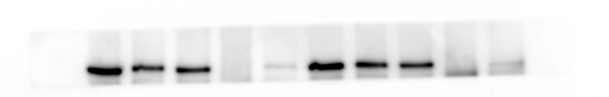

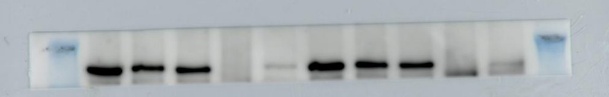


α-Su(Hw)


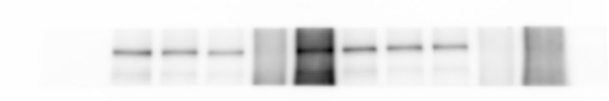

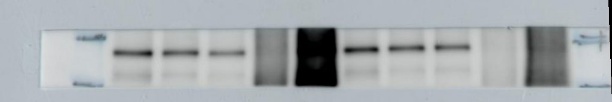


α-Cul4 (rabbit)


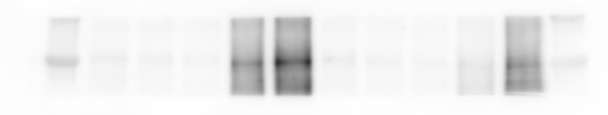

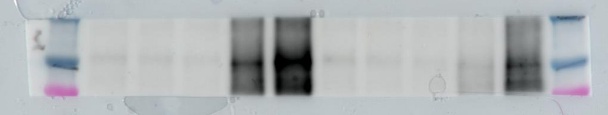


α-Mod(mdg4)67.2


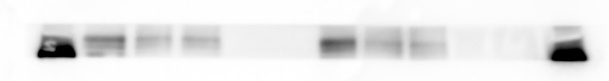

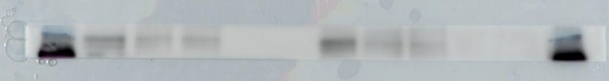


α-LaminB


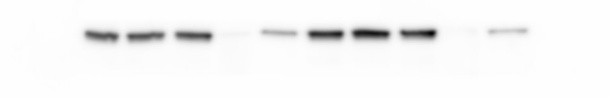

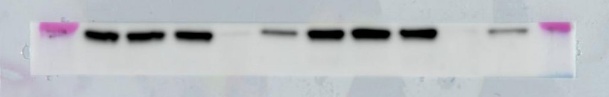


α-Polycomb


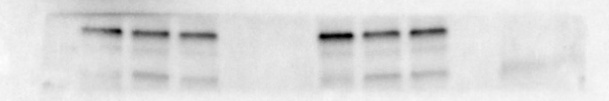

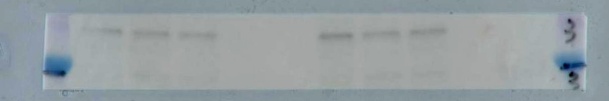


α-Eff (rabbit)


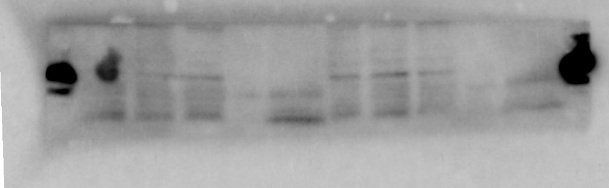

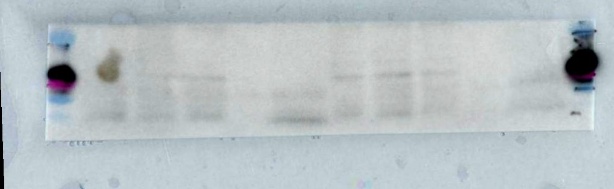


**Corresponding to Figure 2A**

Sample lanes (left to right): Marker, *act5C-Gal4*, *act5C-Gal4, act5C::su(Hw)^RNAi^, act5C::su(Hw)^RNAi^,* *act5C::cul4^RNAi^, act5C-Gal4, act5C::cul4^RNAi^, act5C::eff^RNAi^, act5C-Gal4, act5C::eff^RNAi^,* Marker

α-CP190


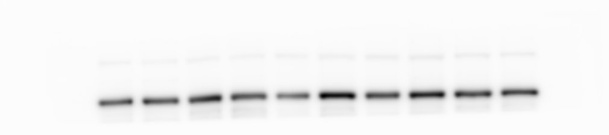

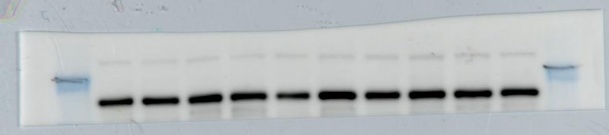


α-Cul4 (mouse)


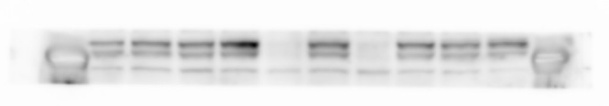

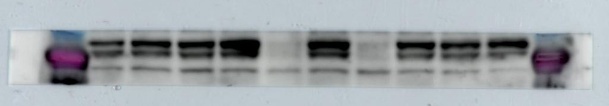


α-Tubulin


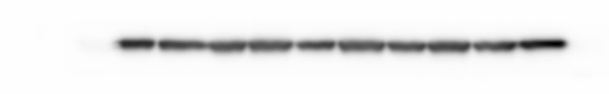

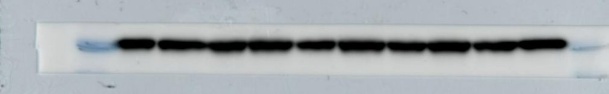


α-Eff (mouse)


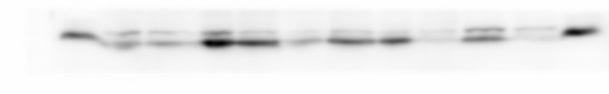

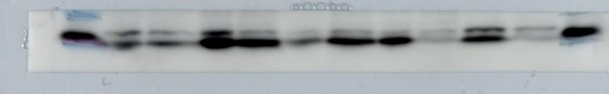


**Corresponding to Figure 5A**

Sample lanes correspond to labeling in main figure.

α-CP190


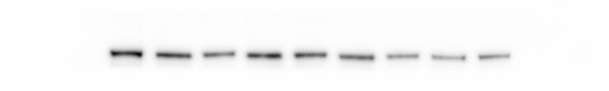

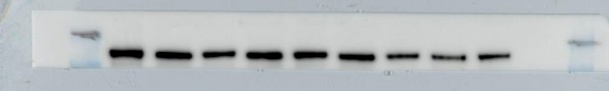


α-Su(Hw)


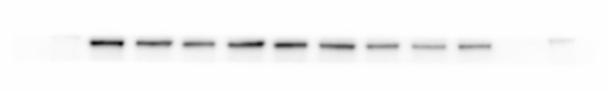

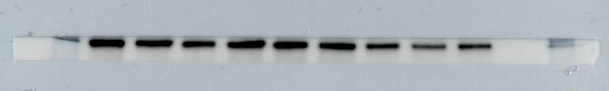


α-Cul4 (mouse)


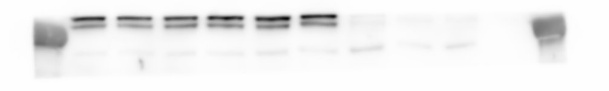

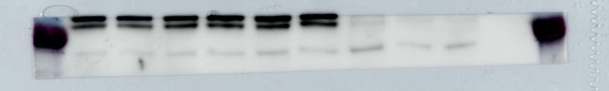


α-LaminB


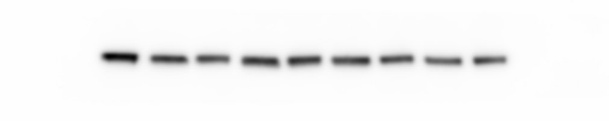

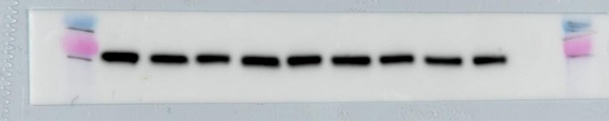


α-Tubulin


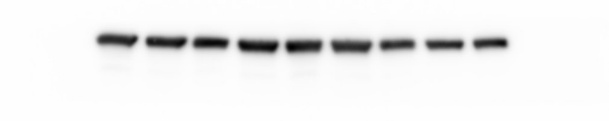

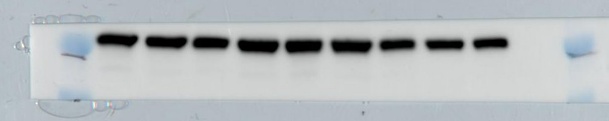


α-Eff (mouse)


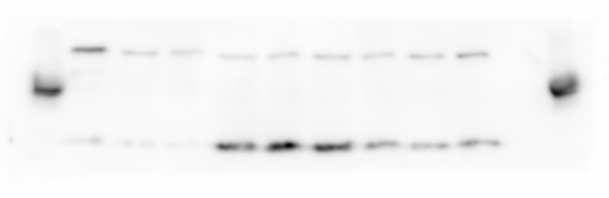

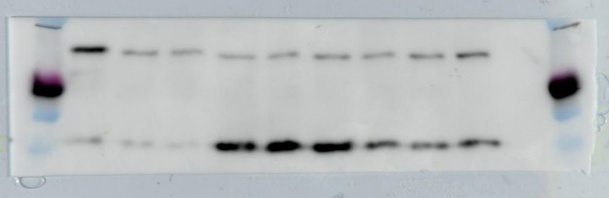


**Corresponding to Additional file 1: Fig. S2B**

α-Cul4 (rabbit)

Sample lanes (left to right): Marker, *mcherry^RNAi^*, *cul4^RNAi^*, *mcherry^RNAi^, cul4^RNAi^*


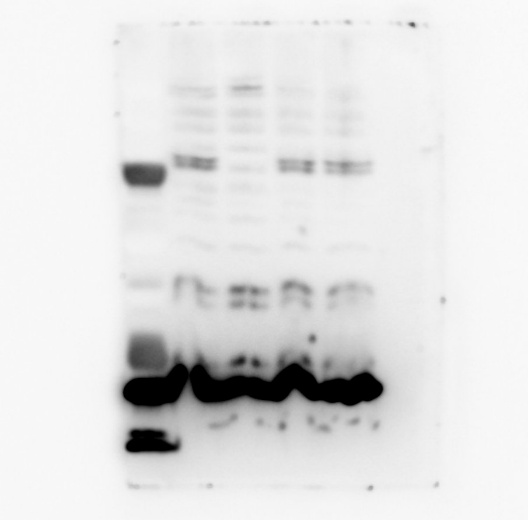

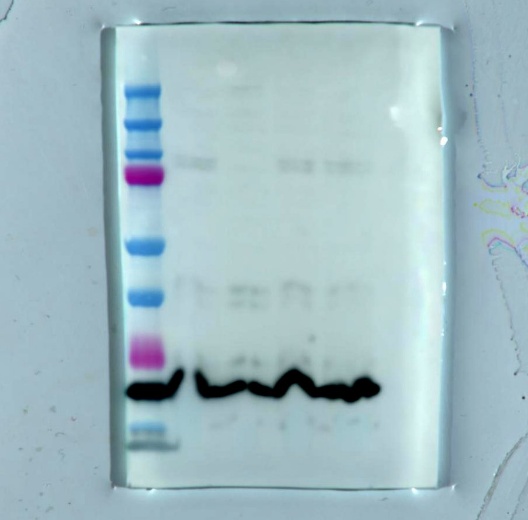


α-Cul4 (mouse)


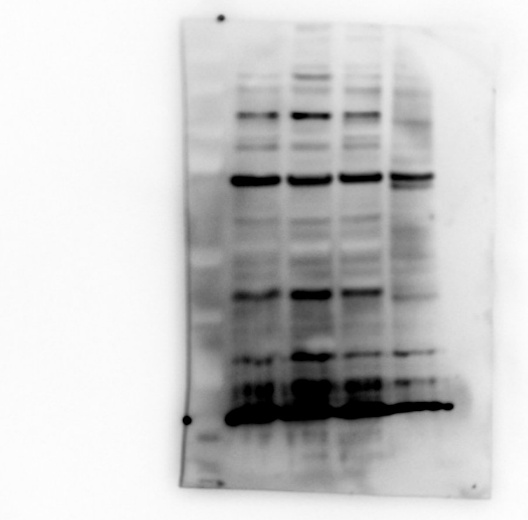

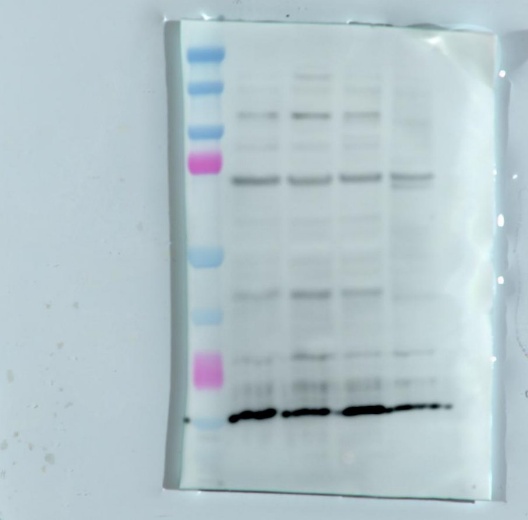


α-CP190


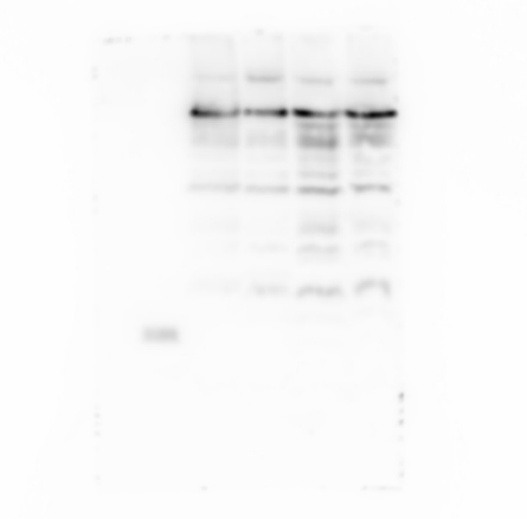

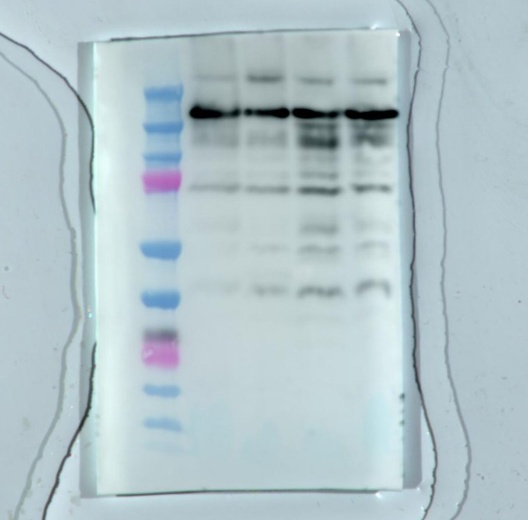


**Corresponding to Additional file 1: Fig. S2E**

Sample lanes (left to right): Marker, *mcherry^RNAi^, cp190^RNAi^, su(Hw)^RNAi^, mod(mdg4)67.2^RNAi^, mcherry^RNAi^, topors^RNAi^, mcherry^RNAi^, topors^RNAi^,* WT1, WT2, Marker

α-CP190


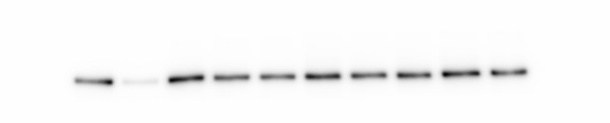

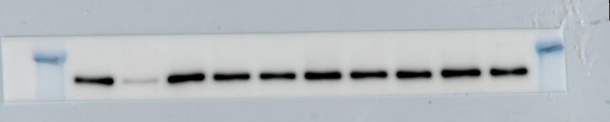


α-Topors


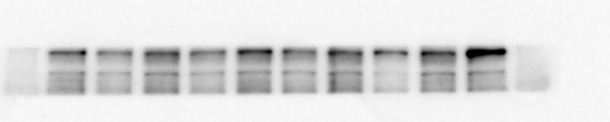

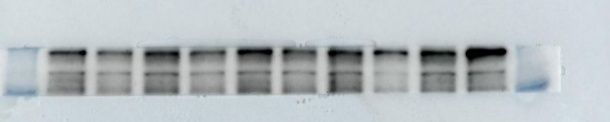


α-Su(Hw)


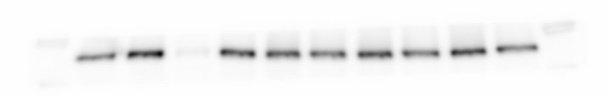

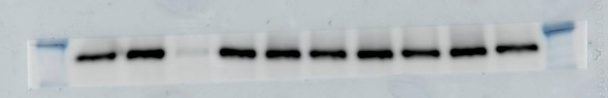


α-Cul4 (mouse)


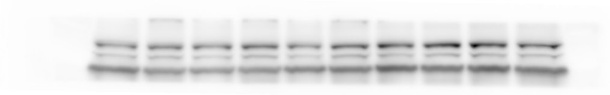

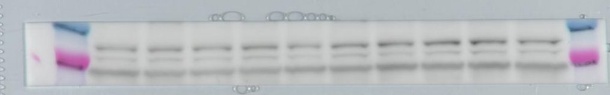


α-Mod(mdg4)67.2


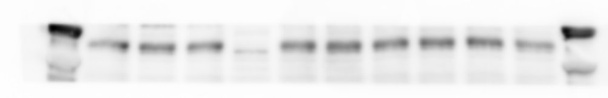

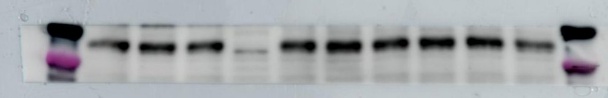


α-Tubulin


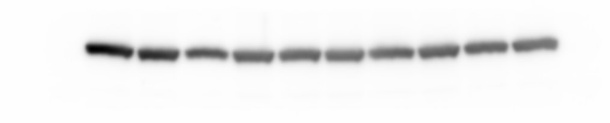

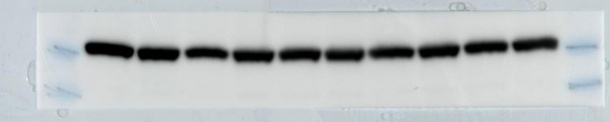


α-Eff (mouse)


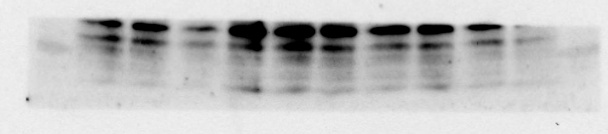

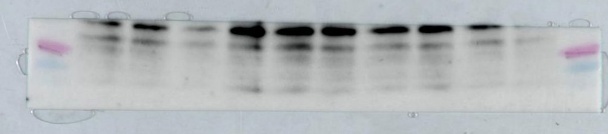


**Corresponding to Additional file 1: Fig. S2G**

Samples (left to right): anti-CP190 IP lanes as labeled in Fig. S2G and nuclear extract, supernatant of IgG IP, supernatant of anti-Eff IP, IgG IP, anti-Eff IP

α-CP190


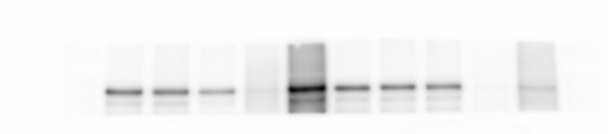

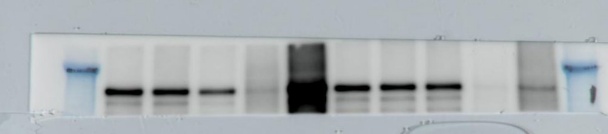


α-Cul4 (rabbit)


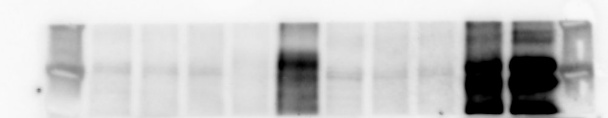

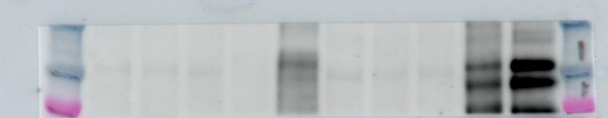


α-LaminB


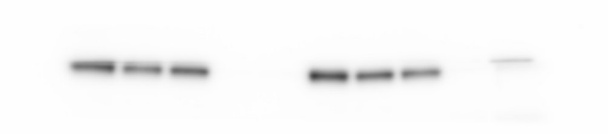

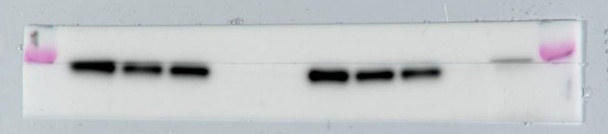


α-Polycomb


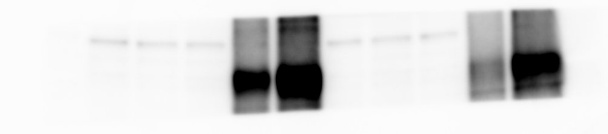

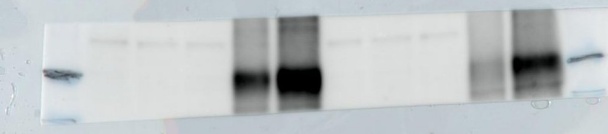


α-Eff (rabbit)


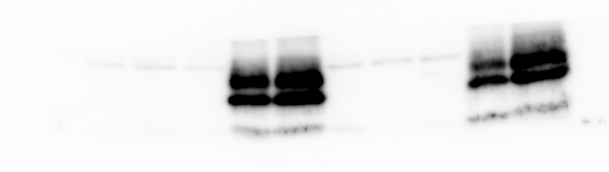

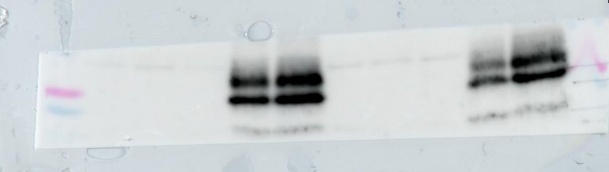

Supplement: Supplementary file 3 — Additional file 3. Original western blot images. [file 12915_2026_2596_MOESM3_ESM.docx]
